# Predicting the future of urban ecological resilience in China’s Yellow River Basin: a machine learning approach

**DOI:** 10.1038/s41598-026-54737-0

**Published:** 2026-05-24

**Authors:** Ting Fan, Xiaoyong Li, Chenlu Huang, Guan Huang

**Affiliations:** 1 Institute of Human Geography, College of Tourism, Xi’an International Studies University, Xi’an, 710128 People’s Republic of China; 2 School of Tourism and Culinary, Yangzhou University, Yangzhou, 225009 People’s Republic of China

**Keywords:** predictive modelling, extreme gradient boosting, early warning, regional ecological assessment, countermeasure, clustering analysis, Ecology, Ecology, Environmental sciences, Environmental social sciences

## Abstract

Urban Ecological Resilience (**UER**) is essential for sustainable development, especially within ecologically sensitive regions such as China’s Yellow River Basin (**YRB**). Existing assessments of **UER** often encounter difficulties attributable to extensive regional boundaries and retrospective methodologies, thereby limiting their applicability in policymaking. To address these limitations, this study presents an innovative framework. Initially, 51 cities were classified into seven functional clusters based on ecological and industrial similarities. Subsequently, the **UER** for each cluster was quantified from 2010 to 2024 utilizing the Entropy Weight Method. Projections for **UER** from 2025 to 2027 were generated employing an XGBoost (eXtreme Gradient Boosting) model that integrates temporal features derived from historical data. The findings indicate a concerning decline in **UER** within the traditional heavy industry cluster, alongside fluctuating decreases in the Loess Plateau agriculture and conventional agriculture clusters. Model interpretations identify vulnerable cities and low-performing indicators, such as per capita water resources, environmental protection budgets, and industrial pollution, which are strongly correlated with these predicted declines. Conditional simulation demonstrates that targeted interventions aimed at these indicators have the potential to mitigate adverse trends. This comprehensive approach provides a quantitative, proactive tool for formulating specific strategies to enhance **UER** across diverse regions.

**Supplementary Information:**

The online version contains supplementary material available at 10.1038/s41598-026-54737-0.

## Introduction

Ecological resilience, defined as an ecosystem’s capacity to maintain its fundamental structure and functions under disturbance, extends to urban ecological resilience (**UER**) in complex, human-influenced urban environments^1^. **UER** specifically denotes the ability of urban social–ecological systems to resist, adapt to, and recover from environmental shocks and stresses^2^. Amid escalating global conflicts between societal development and ecological conservation, understanding **UER** is crucial for ensuring the long-term sustainability and functionality of cities^3^.

**Fig. 1:**
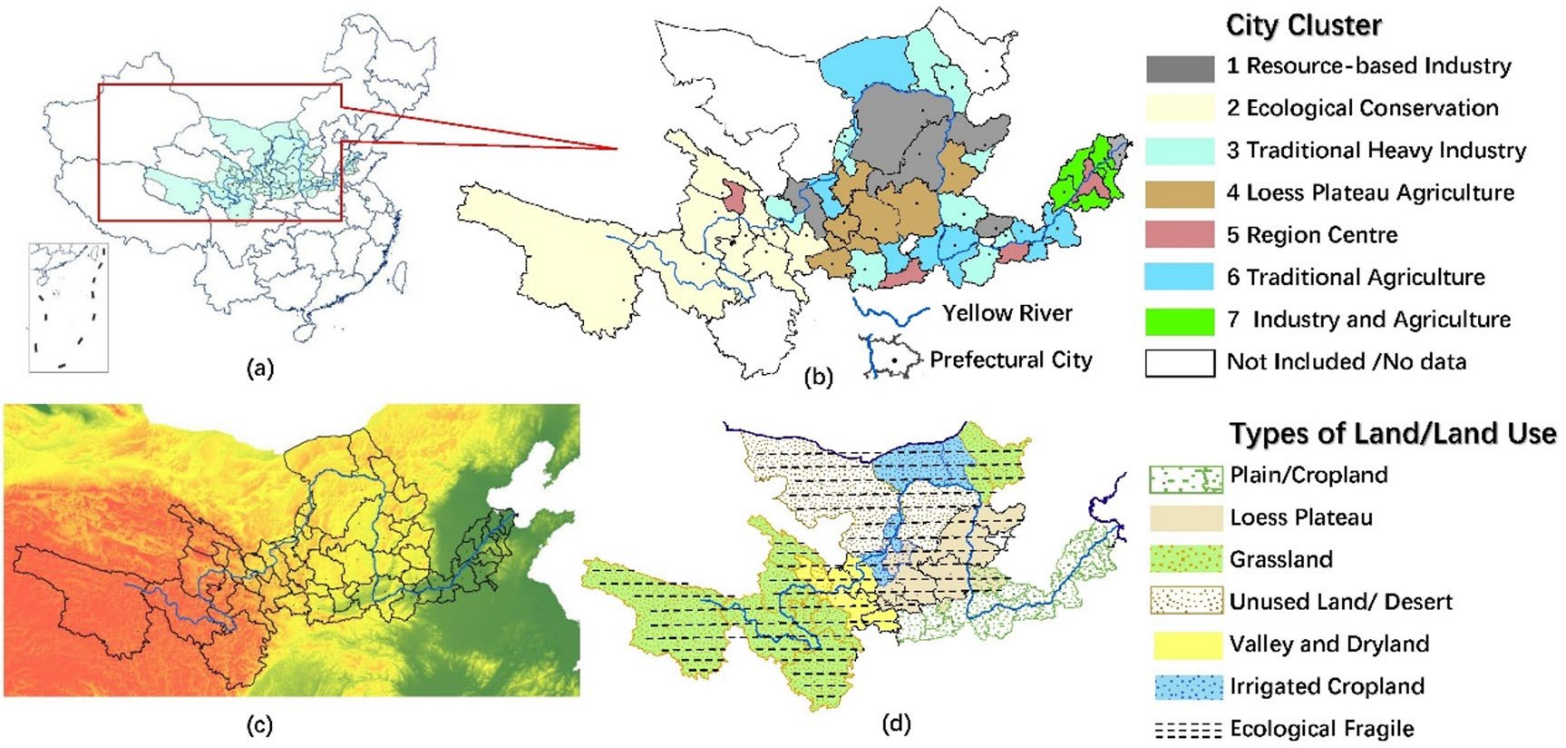
The Yellow River Basin: (**a**) Location and included prefectural cities; (**b**) Typical City Clusters investigated in this study; (**c**) Topographic map; (**d**) Land type and land use patterns.

As a nation undergoing rapid industrialization and urbanization, China confronts significant urban ecological and environmental challenges, amplified by regional geographical variations. The Yellow River Basin (**YRB**, Fig. 1a/b), spanning approximately one-sixth of China’s landmass, is one of the country’s most ecologically fragile zones (Fig. 1c/d). Its Upper and Middle Basins, featuring the Loess Plateau and arid climates^4^, are prone to severe risks such as soil erosion, land degradation, and vegetation loss. Meanwhile, rapid urbanization in the **YRB**, coupled with imbalanced regional economic development, has intensified interconnected social and ecological problems^5^. These include cultivated land degradation, suburban impoverishment from rural-to-urban migration, severe winter air pollution^6^, pronounced heat island effects, and frequent urban waterlogging^7^, all of which undermine urban ecosystem stability.

Recent research on **UER** in the **YRB** has primarily adopted a ‘resistance-adaptation-recovery’ framework for assessment^8^. Evaluation methods commonly use the entropy weight method to derive ecological resilience indices, often combined with techniques such as the Technique for Order Preference by Similarity to Catastrophe Progression Model^9^ or Ideal Solution (TOPSIS)^10^. Driving factors are typically analyzed via geographically weighted regression, identifying socio-economic factors (urbanization rate, GDP per capita, industrial structure) and government policies as positive drivers. Landscape fragmentation, bird diversity^11^, and green coverage also contribute. Spatially, studies using geographical detectors and spatial autocorrelation analysis reveal significant positive spatial correlation (agglomeration) of **UER**, forming distinct ‘high–high’ (HH) and ‘low–low’ (LL) clusters in the **YRB**^10^. High-value areas typically occur in regions with strong ecological foundations, advanced economic development, or cities experiencing lower resource–environmental pressure and substantial governance investment^9,12^. Low-value areas prevail in ecologically fragile zones, traditional resource-based cities, and regions with lagging economic development.

However, existing studies often use provincial^8^ or broad watershed partitions^10^(upper, middle, lower basin) as primary units, which mask critical intra-regional heterogeneity and limit applicability for localized policymaking. For instance, Shaanxi Province includes diverse ecological zones, such as the Loess Plateau, the Guanzhong Plain, and the Southern mountainous forests, making the provincial average **UER** less informative due to obscured internal disparities. Broad watershed divisions span areas larger than provinces, further homogenizing diverse functional zones. While city-level studies provide valuable localized insights^13^, they often overlook the synergistic development potential of regions, limiting support for effective cross-jurisdictional policies. Moreover, existing research relies mainly on static cross-sectional analysis or linear correlation models for retrospective analysis of historical panel data. Although these methods describe past trends and spatial patterns, they inadequately predict future **UER**, as they fail to capture the complex, non-linear relationships and temporal dependencies in urban systems^14^. This limitation impedes effective early-warning systems and targeted countermeasures, such as identifying areas needing pre-emptive interventions to avert future **UER** declines or determining optimal policy levers for specific city groups.

Machine learning (ML) has emerged as a powerful tool in ecological research, distinguished by its superior ability to handle non-linear dynamics compared to traditional methods. For instance, various ML models, such as Support Vector Machine (SVM), Random Forest (RF), K-Nearest Neighbours (KNN), and XGBoost, have been employed to predict groundwater drought conditions based on hydro-meteorological parameters. One study^15^ found that the linear SVM exhibited the best performance in this context. Conversely, in research focused on predicting reference evaporation in semi-arid regions, the non-linear Random Forest model demonstrated superior predictive accuracy, achieving an R² value exceeding 0.94^16^. Beyond environmental applications, the Random Forest model, particularly when combined with SHapley Additive exPlanations (SHAP), has been utilized in urban research to identify the key drivers of economic resilience in Chinese urban areas^17^. Nevertheless, most of these studies trained their models on initial variables without pursuing further refinements, such as feature engineering. Meanwhile, applications in the domain of ecological resilience remain limited.

To address these gaps, we propose a novel framework integrating functional urban clustering with predictive machine learning. We categorize **YRB** cities into seven research clusters based on consistent ecological baseline conditions (e.g., physical geography, land-use types) and industrial development synergies (e.g., primary industries, urbanization level). This functional clustering mitigates unreliability in **UER** assessments from intra-provincial disparities and broad geographical divisions, offering more granular insights than traditional administrative or watershed-level analyses. We then use a curated set of indicators to calculate **UER** for each city cluster from 2010 to 2024 via the entropy weight method. Temporal features are engineered from these time-series data, and an XGBoost model —a robust machine learning algorithm for complex, non-linear patterns—is applied to predict **UER** for each urban cluster from 2025 to 2027. Through Model interpretation, we identify key cities and specific indicators associated with projected **UER** declines. This integrated analysis delivers quantitative, forward-looking, and targeted strategies to support coordinated enhancement of urban ecological resilience across the Yellow River Basin.

## Methodology

### Research flowchart

**Fig. 2:**
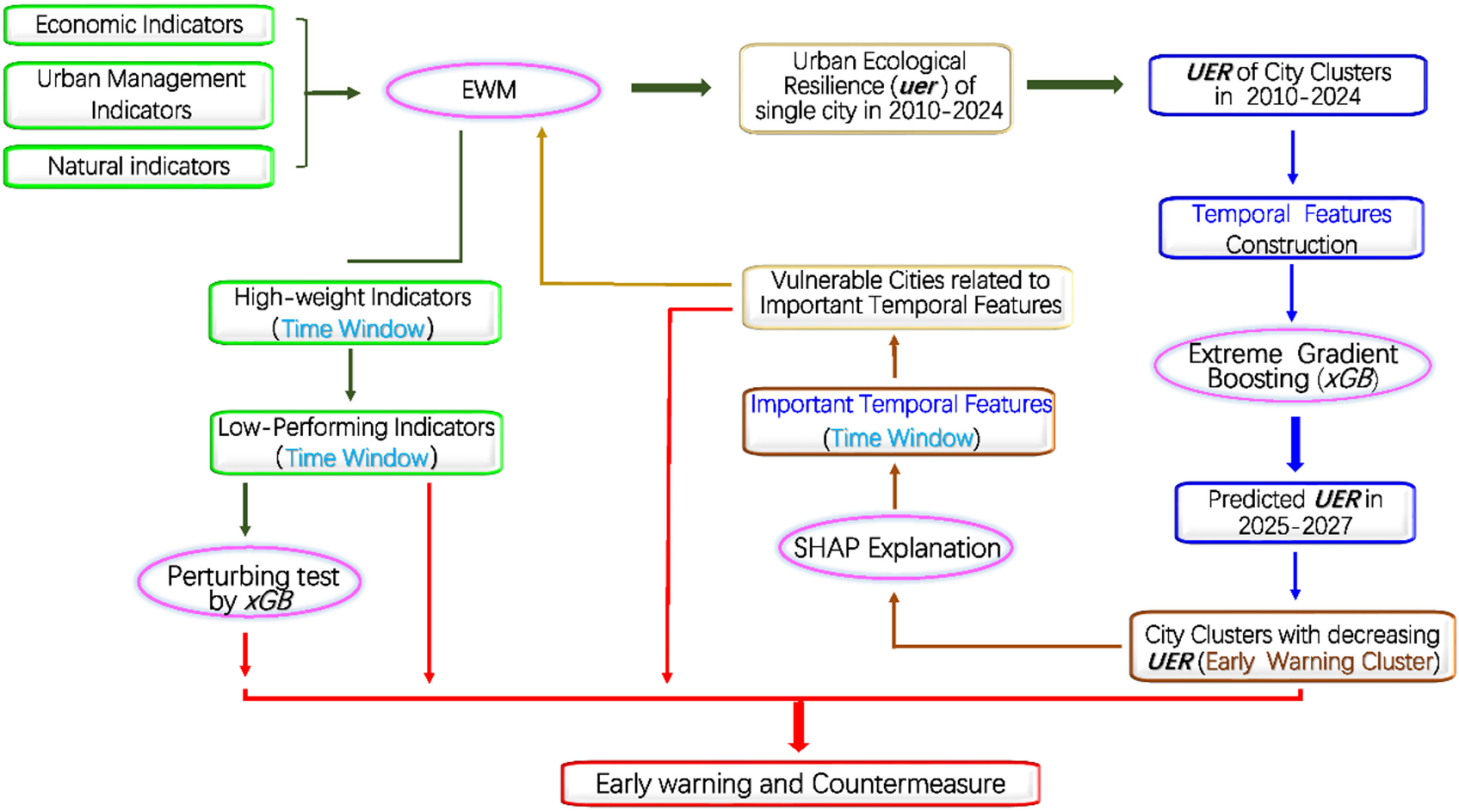
Flowchart of this study.

First, the Urban Ecological Resilience (**UER**) for each city from 2010 to 2024 was computed using the Entropy Weight Method (EWM) based on three major categories of original indicators (Fig. 2). Temporal feature engineering was then applied to the cluster-level **UER** time-series data. An XGBoost model was used to forecast **UER** for each city cluster from 2025 to 2027. For clusters showing a predicted declining **UER** trend (Early Warning clusters), SHAP analysis identified the most influential temporal features and pinpointed specific cities (Vulnerable Cities) contributing to the decline. By examining indicator weights from the EWM output and normalized values in vulnerable cities, key factors (Low-performing Indicators) associated with the **UER** decrease were identified. The potential impact of policy interventions was assessed through simulations, adjusting these Low-performing indicators. Based on this analysis of Early Warning Clusters, Vulnerable Cities, and Low-performing indicators, early warning was issued and targeted, synergistic strategies for enhancing ecological resilience were proposed. Machine learning processes were implemented using Orange 3 (Version 3.36.0) with necessary Python 3.9 code (Supplemental Fig. 2, Text.1, Text.2, Text.3).

### Indicator selection and data sources

Drawing upon prior research focused on the YRB, this study selects 15 indicators across three categories (Table 1): natural conditions (representing resistance), urban management (representing recovery), and economic conditions (representing reconstruction) to comprehensively evaluate urban ecological resilience^12,13,18,19^. Indicators such as high-temperature days and extreme weather events reflect the side effects of urbanization, including the urban heat island effect and climate change vulnerability. Water resources are not just environmental factors but fundamental constraints on urban ecological resilience in the arid **YRB**. For cities with advanced and cleaner industries, growth in **E2** enhances economic capacity and supports environmental reinvestment, thereby exerting a positive influence (+) on **UER**. In contrast, for resource-based cities, traditional heavy industrial cities, or ecologically fragile areas, industrial expansion is frequently linked to higher pollution, greater resource consumption, and increased transformation pressure, resulting in a negative impact (-). Education and scientific-research budgets indicate investment in human capital and technological innovation, crucial for adaptive capacity^20^. Urban greening rate and forest coverage reflect vegetation health and ecological regulatory capacity. Data for 2010–2024 were mainly sourced from the China Urban Statistical Yearbook (2010–2024) and annual Ecological and Environmental Status Bulletins of individual provinces (Supplemental Table 1). The missing values (less than 2%) in specific indicators (**N3**, **U2**, **U3**, **E3**; Supplemental Table 1) were imputed using the mean of the same cluster and year to avoid temporal information leakage. However, these indicators were not included in subsequent K-means clustering, without impacting the results of clustering. Cities with extensive data deficiencies, such as Jiyuan (Henan) and Tongchuan (Shaanxi), with over 30% missing data or absent time series for key indicators, were excluded to ensure data quality. The two cities do not differ substantially in economy and ecology, so their exclusion is unlikely to affect clustering representativeness.

**Table 1:** The indicators for the assessment of urban ecological resilience in this study.

Division	ID	Indicators	Explain	+/−
Natural Indicator (Resistance)	N1	Urban High-temperature Days (days/a)	Days with maximum temperature ≥ 35 °C	**−**
	N2	Water Resource per Capita (m^3^)	Total available renewable water resources (surface and ground) divided by the population.	+
	N3	Extreme Weather (days/a)	Total days of heavy rain/strong wind/hail (details in Supplemental Table 1)	**−**
	N4	Urban Air Quality (days/a)	Number of days with AQI ≤ 100	+
	N5	Desertified Land Ratio (%)	Area ratio of desertified land to total land (%), Soil Degradation Ratio (%) for specific cities	
	N6	Forest Coverage Rate (%)		+
Economic Indicator (Reconstruction)	E1	GDP per capita (thousand CNY/a)		+
	E2	Second Industrial Added Value (billion CNY/a)		**+/−**
	E3	Ratio of Education and Research Budget to GDP (%)	Local Scientific Research and Education Investment	+
	E4	Ratio of Environmental Protection Budget to GDP (%)		+
Urban Management Indicator (Recovery)	U1	Population Density (thousands/per km^2^)	Population per km^2^ of built-up area	**−**
	U2	Industrial Wastewater Discharge (tons per million GDP)		
	U3	Utilization rate of Industrial Solid Waste (%)	Ratio of recycled Industrial Solid Waste to total produced	+
	U4	Urbanization Rate (%)	Ratio of urban built-up area to total area	
	U5	Urban Green Coverage (%)		+

### Classification of city clusters

Using K-means clustering and referencing regional planning and economic geography documents^21,22^, the 51 prefecture-level cities in the **YRB** (only those with administrative centers within the basin) were categorized into seven functional clusters. K-means was applied to a standardized feature set describing baseline ecological, socio-economic, and geographic attributes (Supplemental Table 2), with continuous variables Z-score standardized and categorical variables as vectors (Supplemental Table 3). K values from 5 to 11 were tested, and K = 7 was selected based on Silhouette criteria and stability (50 random initializations; Supplemental Table 4):

Cluster 1 (Resource-Based Industry): Cities focused on energy and resource extraction (e.g., Baiyin, Yulin, Ordos, Dongying), with mining-related pollution and arid ecological baselines. These cities share economic structures and environmental pressures despite lacking geographical contiguity.

Cluster 2 (Ecological Conservation Area): Areas designated for conservation, mainly in eastern Gansu and Qinghai, with fragile ecosystems and high ecological value. Economies rely on animal husbandry and tourism.

Cluster 3 (Traditional Heavy Industry): Cities with entrenched heavy industry (e.g., Baotou, Taiyuan, Linfen, Luoyang), where the Secondary Industry exceeds 60% of GDP, are facing economic downturns, pollution, and transformation pressures.

Cluster 4 (Loess Plateau Agriculture): Loess Plateau cities focused on agriculture, grappling with soil erosion and water scarcity (e.g., Yan’an, Tianshui, Guyuan, Lvliang). This is the least developed YRB area, where agriculture and industrialization are constrained by fragile ecological capacity.

Cluster 5 (Regional Central Cities): Major economic and political hubs (e.g., Xining, Xi’an, Zhengzhou, Lanzhou, Jinan), driven by the tertiary Industry and advanced manufacturing.

Cluster 6 (Traditional Agriculture): Cities dominated by agriculture, distinct from the Loess Plateau with better water supply (e.g., Zhongwei, Bayan Nur, Xianyang, Weinan, Kaifeng, Heze). They face demands for industrialization to drive economic growth.

Cluster 7 (Industry and Agriculture): developed Cities with advanced industrial and agricultural sectors, mainly in Shandong Province’s Lower Basin (e.g., Tai’an, Liaocheng, Zibo, Binzhou, Dezhou).

K = 7 was selected to achieve good cluster separation and internal stability, thereby reducing feature dimensionality and research complexity while enhancing distinctiveness and policy value. It avoids issues from amalgamating cities with significant ecological and industrial differences into broad provincial or watershed divisions.

### The calculation of urban ecological resilience

The urban ecological resilience index (simplified as **UER**) of an individual city was calculated using the Entropy Weight Method (**EWM**)^10,23^, and the original indicators were normalized by:

1 $$r_{{ij}} = \frac{{x_{{ij}} - \min x_{{ij}} }}{{\max x_{{ij}} - \min x_{{ij}} }}$$

2 $$r_{{ij}} = \frac{{\max x_{{ij}} - x_{{ij}} }}{{\max x_{{ij}} - \min x_{{ij}} }}$$

Formulas (1) and (2) are used for positive attributed indicators (urban green coverage, etc.) and negative indicators (extreme weather days, etc.), respectively. *x*_ij_ represents the value of indicator *j* for city *i* (*i* = 1, 2, …, 51; *j* = 1, 2, …, 15), *r*_ij_ is the standardized value, and max*x*_ij_ and min*x*_ij_ indicate the maximum and minimum value of the *j*-th indicator. Weights were computed per year (year-varying) to capture the dynamic changes in the relative importance of indicators over time. This approach ensures that the **UER** assessment tracks trends and associated factors, rather than emphasizing retrospective comparison within the time series between different clusters. For each indicator *j*, the proportion *p*_ij_ of the *i*-th city to the sum of that indicator was computed:

3 $$p_{{ij}} = \frac{{r_{{ij}} }}{{\sum\nolimits_{{i = 1}}^{{\mathrm{n}}} {r_{{ij}} } }}\quad {\mathrm{n}} = {\mathrm{51}}$$

The entropy value (*e*_j_) for indicator *j* was computed using the formula:

4 $$e_{j} = \frac{{\sum\nolimits_{{i = 1}}^{n} {p_{{ij}} \ln p{}_{{ij}}} }}{{\ln n}}$$

The objective weight (*w*_j_) for each indicator was obtained by normalizing its divergence coefficient (1-*e*_j_):

5 $$w_{j} = \frac{{\left( {{\mathrm{1}} - e_{{\mathrm{j}}} } \right)}}{{\sum\nolimits_{{j = 1}}^{n} {\left( {{\mathrm{1}} - e_{{\mathrm{j}}} } \right)} }}\quad \left( {\sum\limits_{{j = 1}}^{n} {w_{j} = 1} } \right)$$

The weights are applied to the normalized data to obtain a score as ***uer***_i_ for city *i*:

6 $${\user2{uer}}_{i} = \sum\limits_{{j = 1}}^{n} {w_{j} \times r_{{ij}} }$$

Since most indicators were already averaged across GDP, population, or area, the total **UER** of a city cluster was calculated as the arithmetic mean of the ***uer*** of its individual constituent cities, as applied in previously published documents^10,12,18^.

The trend in cluster-level **UER** computed using alternative standardized approaches (Z-scores) and more sophisticated weighting techniques (TOPSIS) demonstrates consistency. Given that the model mainly forecasts future **UER** variations, the simplest entropy method is appropriate here. It also reduces the influence of complex processing on temporal features, allowing the model to learn underlying patterns more realistically.

### Temporal feature engineering and prediction

To account for temporal lag effects, new temporal feature variables were constructed from the **UER** time-series data of each city cluster (Table 2). These included lagged **UER** values over the past three years, their rolling growth rates and averages within this window, and year-on-year change rates^24^. The three-year window balances data length, overfitting reduction, and short-to-medium-term policy planning cycles.

**Table 2:** Temporal features and their construction for city clusters in model training.

Temporal feature	Temporal variable	Definition (t is the current year)
Lag Effect	Lag_1, Lag_2, Lag_3	***UER*** of t-1, t-2, and, t-3, respectively
Rolling Feature	Roll _std_, Roll_mean_	Standard deviation and mean value of ***UER*** from t-1, t-2, t-3
Difference Feature	Diff_1, Diff_2, Diff_mean_	Difference in ***UER*** between neighbouring years, t-1, t-2, t-3, and their mean, respectively

Since nonlinear models can better capture complex urban-environment dynamics for forecasting ecological variables^25,26^, this study used the nonlinear XGBoost model^27^ rather than linear methods like ARIMA (Autoregressive Integrated Moving Average)^28^. Samples were built with a three-year sliding window on the cluster level (details in Supplemental Table 5): the input variables for each sample consisted of the **UER** and its corresponding temporal features from three consecutive years (t-1, t-2, t-3), and the target variable was the **UER** of the year to be predicted (t, or t + 1, or t + 2). The models (**M1**, **M2**, **M3**) were trained independently to predict **UER** for t, t + 1, and t + 2, respectively. The period from 2010 to 2019 served as the training set, taking Model **M1** as an example, the samples span from the window 2010–2012 (predicting 2013) to the window 2016–2018 (predicting 2019). The samples of validation set (2020–2022) and test set (2023–2024) were constructed similarly, respecting the temporal order. The fine-tuning of hyperparameters was performed via grid search (see Supplemental Table 6) on the validation set and not nested within the Cross-Validation set, preventing the potential leakage of future data. Considering the total 7 clusters, for Model **M1 (**Supplemental Table 5), the training set included 49 samples (2013–2019), the validation set used 21 samples (2020–2022), and the test set had 14 samples (2023–2024); the samples of **M2**/**M3** are by parity of reasoning.

The uncertainty the prediction was evaluated by performing a Monte Carlo simulation (100 iterations). In each iteration, the three most recent annual **UER** values (used as model input) were independently perturbed by adding random noise drawn from a normal distribution with zero mean and a standard deviation equal to the standard deviation of the year-to-year **UER** changes for that cluster over the historical period (2010–2024). From each perturbed three-year sequence, the temporal features were recalculated and fed into the models (**M1/M2/M3**) to obtain a prediction. The 2.5th and 97.5th percentiles (95% confidential interval, 95% CI) of the resulting 100 predictions constituted the lower and upper bounds of the prediction interval.

For comparison, two baselines were employed: ARIMA and an L2 regression (Ridge) linear model. The ARIMA model was trained using **UER** data from 2010 to 2022. The L2 regression model, which specifically utilized the same temporal features as XGBoost, had its optimal regularization strength evaluated via grid search. The predictions on 2023–2024 were compared to real **UER** (EWM-calculated) and XGBoost (**M1**).

### Explanation of results and policy suggestions

The temporal features lack direct ties to specific indicators, limiting the interpretability of models. To trace the associations within the model back to actionable policy levers, we adopted the following procedures:

- Early Warning Cluster: Clusters with declining **UER** forecasts for 2025–2027 were targeted for further analysis;
- Important Temporal Features: SHAP analysis^29^ of the XGBoost model identified features with strong association to the predicted **UER** declines of the Early Warning Cluster;
- Vulnerable Cities: For the Early Warning Cluster, cities whose historical ***uer*** negatively impacted the Important Temporal Features (which were calculated from cluster-level UER data, originally calculated from city-level UER) were evaluated as Vulnerable Cities that contributed to the cluster’s decline;
- High-weight Indicators: For Vulnerable Cities, annual weights of 15 indicators from 2022 to 2024 (Formula 5) were ranked in descending order (*w*_1_, *w*_2_, …,*w*_j_). The top n indicators whose cumulative sum equaled or exceeded 0.6 (*w*_1_ + *w*_2_+*w*_3_+…+*w*_n_≥0.6) were selected. Those demonstrating a consistent year-on-year weight increase were categorized as High-weight Indicators.
- Low-performing Indicators: For High-weight Indicators, the product of weights and normalized values (*w*×*r*) was computed (Formula 6); indicators contributing less than 5% of the total *w*×*r* and exhibiting a decreasing trend were identified as Low-performing.

Based on the identified Vulnerable Cities and Low-performing Indicators, combined with the local economic and ecological status, corresponding policy countermeasures can be suggested to improve **UER**.

A simulation study^30^ was performed to assess the effectiveness of policy countermeasures on clusters’ **UER** by intervening on Low-performing indicators in Vulnerable Cities. In the intervention scenario, positive indicators were assumed to increase by 10% annually in 2025 (from 2024) and 2026 (from 2025), while negative indicators decreased equivalently (Supplemental Table 8). Conversely, without intervention, positive indicators were assumed to decrease by 5% annually, and negative ones increased by 5%. These 5% and 10% change ranges are used for illustrative conditional scenario analysis. These simulations project potential outcomes based on the model’s associations and are not a direct causal proof of policy effectiveness. After updating these indicators, the **UER** and related temporal features for 2024–2026 were recalculated. Based on these recalculated temporal features, the **UER** of 2027–2029 was then predicted by models (**M1**, **M2**, **M3)** and discussed.

### Robustness

This study used Time Series Cross-Validation (**CV**) to evaluate model generalization and prevent overfitting^31,32^. For model **M1** (one-year forecast), the data from 2010 to 2021 were chronologically partitioned into six training-test pairs (Supplemental Table 7). The training set spanned 2010–2015 to predict 2016, then 2010–2016 to predict 2017, and so on, up to training on 2010–2020 to predict 2021. For model **M2** (two-year ahead forecast), five pairs were used (e.g., training 2010–2015 to predict 2017, up to training 2010–2019 to predict 2021). For model **M3** (three-year ahead forecast), four pairs were used (e.g., training 2010–2015 to predict 2018, up to training 2010–2018 to predict 2021). Each pair in the **CV** also included 7 samples corresponding to the 7 clusters. This rolling-origin approach ensured that the temporal order of the data was preserved and that the model was tested on unseen future data at each step.

For robustness against perturbations^33^, 10% of the city-year data were randomly altered. One scenario applied + 30% to high-temperature days and − 30% to per capita water resources for extreme climate events. Another scenario simulated economic shocks in industry-dominated city clusters by reducing industrial added value and wastewater discharge volume by 20% to mimic structural shocks analogous to the COVID-19 pandemic. For each scenario, perturbations on raw indicator values and the subsequent normalization were kept within realistic and plausible limits (e.g., no negative water resources). Then, the indicator weights and **UER** were recalculated, temporal features reconstructed, and the trained models used for prediction. Performance was evaluated using Mean Absolute Error (**MAE**), Mean Squared Error (**MSE**), Root Mean Squared Error (**RMSE**), and the Coefficient of Determination (**R**²).

## Results

### **UER** of the Yellow River Basin in 2010–2024

**Fig. 3:**
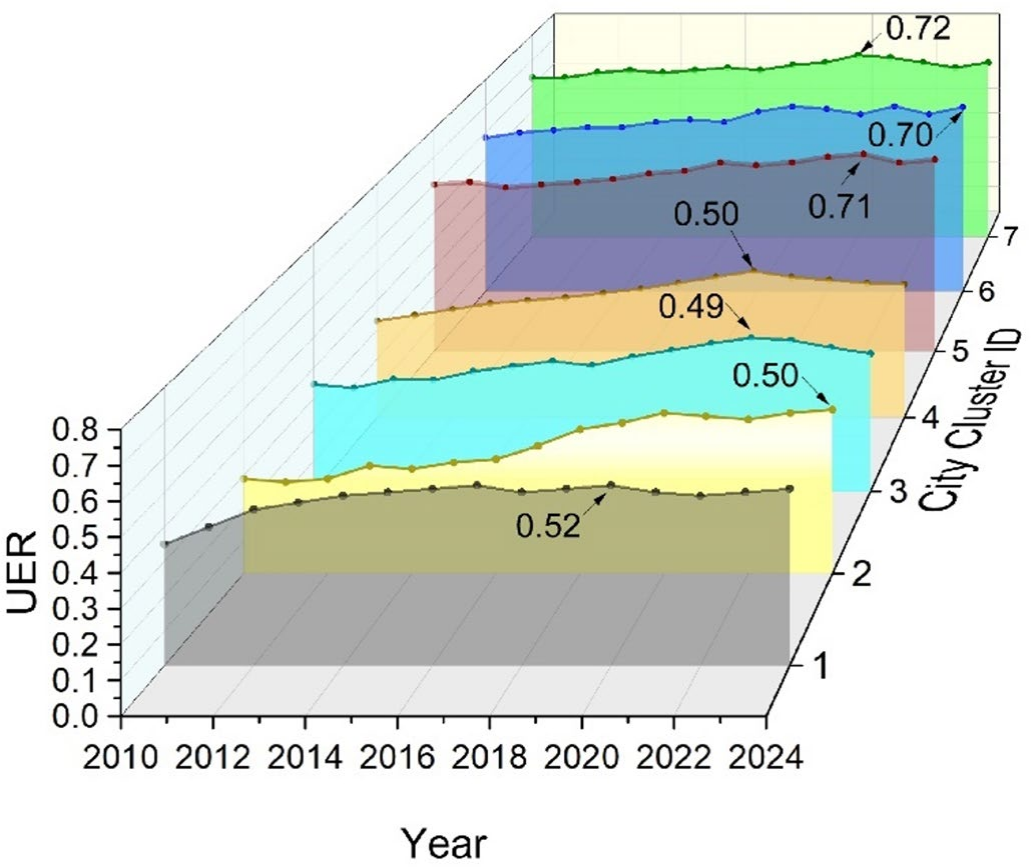
The urban ecological resilience (**UER**) of city clusters of the Yellow River Basin from 2010 to 2024 (value with arrow indicates the highest **UER** of each cluster in the studied period).

From 2010 to 2019, **UER** across **YRB** city clusters generally showed a fluctuating upward trend (Fig. 3)^18^. Post-2020 COVID-19 outbreak, most clusters declined due to economic disruptions and fiscal reallocations^34^, with recovery post-2023 as activities resumed.

**Cluster 1** (Resource-Based Industry) increased gradually, fluctuating with resource prices and industrial transformations. **Cluster 2** (Ecological Conservation Areas**)** achieved sustained growth from a low base, aided by central ecological compensation and industrialization limits. **Cluster 3 (Traditional Heavy Industry)** improved slowly from a low point, driven by post-2016 governance; pandemic shutdowns briefly boosted **UER** via reduced pollution^35^, but 2023 emission surges caused declines, revealing governance gaps. **Cluster 4** (Loess Plateau Agriculture) trended upward from restoration projects but stagnated during COVID-19 due to slowed investments. **Cluster 5** (Regional Central Cities**)** maintained high resilience via policy support, environmental investment, and service economies. **Cluster 6 (Traditional Agriculture)** stayed stable within a narrow range, buffered from industrial shocks by agricultural steadiness. **Cluster 7** (Industry and Agriculture), the most developed, held the highest **UER**, supported by economic growth and advanced governance.

Compared to provincial assessments, this study disaggregates Shaanxi: northern energy cities (e.g., Yulin) in **Cluster 1**, central agricultural ones (e.g., Xianyang) in **Cluster 6**, and Xi’an in **Cluster 5**. This reveals Shaanxi’s **UER** to be a composite of transformation pressures, agricultural stability, and policy-driven growth, which provincial averages tend to mask. The annual standard deviations of city-level UER within each cluster were consistently ≤ 10%, validating the accuracy of K-means clustering.

### Prediction of future **UER** and model comparison

**Fig. 4:**
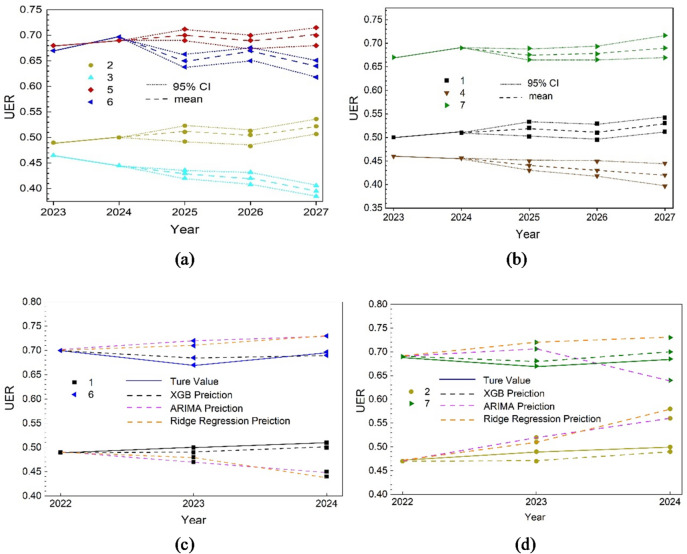
The **UER** predicted by XGBoost from 2025–2027 (**a** and **b**, showing predicted values and 95% prediction intervals derived from the model’s uncertainty); (**c**) and (**d**) comparison between XGBoost, ARIMA, and Regularized Linear Regression model in the prediction of **UER** in 2023–2024.

Predictions show **Cluster 3** (Traditional Heavy Industry) is expected to continue declining through 2025–2027 (Fig. 4a), signaling that these cities face profound structural challenges in their industrial transformation. The **UER** of **Clusters 4** (Loess Plateau Agriculture) and **6** (Traditional Agriculture) are projected to experience fluctuating declines (Fig. 4b), linked to water scarcity and pollution pressures. In contrast, the **UER** of **Clusters 1** (Resource-Based Industry) and **5** (Regional Central Cities) maintain stable, slight increases. **Cluster 2** (Ecological Conservation Area) shows the strongest upward trend, affirming the sustained benefits of ‘protection-first’ strategies. **Cluster 7** (Industry and Agriculture) exhibits a fluctuating but overall rising trend, suggesting that its strong economic base enables continued investment in environmental management. Comparison tests (Fig. 4c/d), conducted on representative Clusters **1**, **2**, **6**, and **7** within the 2023–2024 dataset, revealed no significant difference between XGBoost predictions and actual **UER** (*p* ≥ 0.90), unlike ARIMA (0.29 ≥ *p* ≥ 0.13) and Ridge (0.33 ≥ *p* ≥ 0.10). For **Cluster 7**, ARIMA even predicted an opposite trend, indicating the limitation of its linear character on complex temporal-series data. The comparative results for other Clusters are consistent with this observation, indicating that XGBoost is more accurate and reliable than linear or regularized linear models.

### Explanation of the prediction results

Projected **UER** declines in **Cluster 3**, and fluctuating ones in **Clusters 4** and **6**, are associated with factors such as water scarcity, insufficient environmental investment, and industrial pollution, requiring targeted analysis. For **Cluster 3** (Traditional heavy industry), SHAP on **M1**–**M3** identified Diff_mean_ and Roll_mean_ as key features influencing **UER** (Table 3, Supplemental Table 9). Cities like Shizuishan, Linfen, and Baotou showed the most negative Diff_mean_ and Roll_mean_ values, thus contributing to the cluster’s decline. Weight analysis revealed indicators such as **U2** (industrial wastewater discharge), **N2** (per capita water resources), and **E3** (education and research budget) with cumulative weights > 0.6 in 2022–2024 as High-weight Indicators (Supplemental Table 10). Low-performing indicators (Supplemental Table 9) included: Shizuishan—**N2**, **E4** (environmental protection budget), **N4** (urban air quality); Baotou—**N2**, **E2** (second industrial added value), **U2**; Linfen—**N2**, **U2**, **N4** (urban air quality). These cities and indicators represent the main levers for **UER** improvement in 2025–2027.

**Table 3:** Important temporal features, vulnerable cities, and low-performing indicators in 2022–2024 related to the decrease in predicted **UER** for 2025–2027, extrapolated from the explanation of the Models.

Early warning cluster	Important temporal feature	Vulnerable cities	Low-performing Indicators
3 Traditional Heavy Industry	Diff_mean_ Rol_mean_	Shizuishan (6402) ^*^	**N2** Water Resource per Capita **E4** Environmental Protection Budget **N4** Urban Air Quality
		Linfen (1410)	**N2**; **E2** Second Industrial Added Value; **U2** Industrial Wastewater Discharge; **N4**
		Baotou (1502)	**N4**; **E2** ; **U2**
4 Loess Plateau	Diff_mean_ Lag_3	PingLiang (6208)	**N2; N5** Desertified Land Ratio; **E4;**
		Guyuan (6404)	**N2;E4;N5**
		Qingyang (6210)	**N2;N5;U2**
6 Traditional Agriculture	Roll_mean_ Diff_mean_	Zhongwei (6405)	**N2**; **N5**; **E4**;
		Heze (3717)	**N4; E3** Ratio of Education and Research Budget; **U2**
		Yuncheng (1408)	**N2**; **E3**; **U2**;

*The geographical location of cities can be found in Supplemental Fig. 1 through the four-digit administrative code in parentheses.

Within **Cluster 4**, cities such as Qingyang, Pingliang, and Guyuan face severe water scarcity and have less developed economies. Although past ecological restoration efforts yielded improvements, recent reductions in environmental investment have stalled this momentum, making them vulnerable to renewed degradation. For **Cluster 6**, cities such as Zhongwei, Heze, and Yuncheng are constrained by limited per capita water resources. They are also attempting to modernize agriculture while simultaneously pursuing industrialization, leading to increased wastewater discharge and pollution pressures.

### Robustness of models

**Table 4:** The error parameters for evaluating the robustness of Models (**CV**: cross-validation).

Data set	MAE	MSE	RMSE	*R*²
Training	0.051 ± 0.003	0.0024 ± 0.003	0.052 ± 0.003	0.94 ± 0.050
Validation	0.065 ± 0.002	0.0045 ± 0.004	0.062 ± 0.003	0.90 ± 0.037
Test	0.063 ± 0.004	0.0047 ± 0.002	0.064 ± 0.004	0.89 ± 0.069
CV	0.056 ± 0.002	0.0036 ± 0.001	0.061 ± 0.004	0.90 ± 0.063
Extreme Weather	0.068 ± 0.004	0.0052 ± 0.003	0.067 ± 0.005	0.87 ± 0.065
Economic Impact	0.073 ± 0.005	0.0061 ± 0.004	0.069 ± 0.004	0.86 ± 0.071
Std_*mean*_	0.008	0.0013	0.006	0.028

The trained models demonstrated stable errors and strong explanatory power across the training, validation, test, and cross-validation (**CV**) sets (Table 4). Specifically, the **MAE** ranged from 0.051 to 0.065, the **MSE** varied from 0.0024 to 0.0047, and the R**MSE** fell within 0.052 to 0.064. The **R²** declined slightly from 0.94 on the training set to 0.89 on the test set, indicating good generalization with only minor attenuation on unseen data, even with the inclusion of the volatile COVID-19 period. Under simulated extreme weather or economic shocks, errors remained acceptable (**MAE** 0.068–0.073, **MSE** 0.0052–0.0061, **RMSE** 0.067–0.069), with **R**² values of 0.86–0.87. The standard deviations (*Std*_mean_) for each parameter were only approximately 10% to 15% of their respective mean values across the different datasets and scenarios, which further highlights the robustness and repeatability of the model.

### Policy implications

1. **Cluster 3**’s predicted continuous decline from 2025 to 2027 highlights transformation challenges in Vulnerable cities like Shizuishan, Linfen, and Baotou, targeting indicators such as per capita water resources, environmental budgets, and air quality. Shizuishan should boost environmental investment and curb coal expansion to improve air quality. Baotou must advance industrial wastewater treatment and steel emissions control. Linfen should prioritize water pollution control and phase out polluting enterprises.
  Conditional simulation tests (Fig. 5) demonstrate that if vulnerable cities effectively implement the proposed countermeasures in 2026 and achieve a 20% improvement in low-performance indicators (based on 2024 values), then, relative to the scenario without intervention—in which these indicators decline by 5% annually—the **UER** of **Cluster 3** could potentially increase by approximately 10% in 2027. The decline observed in 2028–2029 is expected to decelerate further and may even reverse. In the absence of intervention and with indicators allowed to deteriorate, the **UER** will experience a sustained and considerable decline. Nonetheless, economic stagnation and policy pressures from heavily polluting industries constitute primary uncertainties for these cities.
  **Fig. 5:**
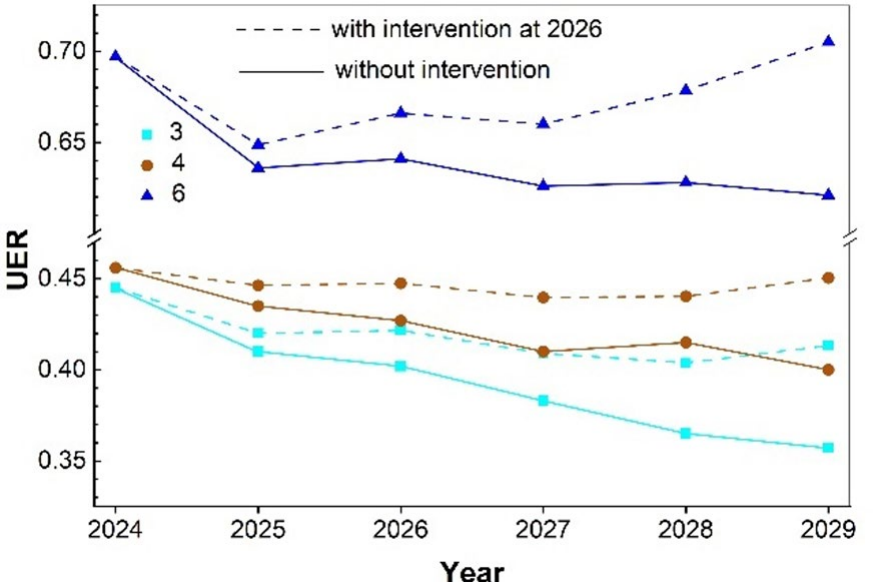
**UER** forecast for 2027–2029 with low-performing indicators improved by 10% (with intervention) or deteriorated by 5% (without intervention) annually for 2025 and 2026 based on 2024.
  Notably, improving a single indicator in a single city yielded only minimal **UER** gains, underscoring that enhancing regional resilience requires comprehensive, integrated participation from the most vulnerable cities within a cluster.
2. **Cluster 6** is projected to experience a fluctuating **UER** decline, driven by pressures on per capita water resources, desertification, air quality, and industrial wastewater discharge in key cities such as Zhongwei, Heze, and Yuncheng. These cities are expected to undergo industrialization, but they must reject relocated polluting industries from the East Coast regions. Zhongwei should match agriculture to water availability, integrating water-saving irrigation and desertification control. Middle- and Lower-basin cities such as Yuncheng and Heze must restrict polluters, invest in agricultural innovation, and adopt water-saving measures for agricultural modernization. Cluster-wide, basin water allocation and coordinated water-saving agriculture can fund protection.
  If the above policies are well implemented and all low-performing indicators achieve the preset improvement by 2026, the UER of Cluster 6 may potentially stabilize or rise from 2027 to 2029 (Fig. 5b), potentially exceeding the 2024 level in 2029. If no active intervention is taken, the **UER** will experience a fluctuating decline from 2027 to 2029. Whether new technologies can enhance agricultural efficiency and farm profits remains uncertain.
3. The **UER** of **Cluster 4** improved due to central government investment on the Loess Plateau in the 2010–2020 decade, but declined as financial support waned in the post-COVID-19 era. Cities such as Qingyang, Pingliang, and Guyuan are primarily affected, facing severe water scarcity and desertification. Pingliang should shift to an ‘ecological economy’ blending water-saving agriculture and tourism. Qingyang and Guyuan must address water resources by restricting highly water-consuming industries, safeguarding environmental budgets, and implementing slope-stabilization projects to control soil erosion. Given the region’s aridity and extensive wasteland, developing eco-friendly industries like photovoltaic and wind power presents an alternative pathway for boosting GDP and financial revenue, thereby providing a sustainable funding source for continued ecological management.
  Under active improvement in 2026, the **UER** of **Cluster 4** can remain stable within the period of 2027–2029 compared to the fluctuating decline without intervention. Given China*’s* sluggish economic climate, this outcome is deemed acceptable for the fragile Loess Plateau region. Without intervention, intensified soil erosion may pose greater safety risks to the middle and lower reaches of the **YRB**. In recent years, the northward shift of the summer rain belt in China has helped alleviate the water resource problems of these agricultural cities, provided that soil and water conservation and water conservancy facilities keep pace.
4. **Cluster 1** (Resource-Based Industry) and **Cluster 5** (Regional central cities) are projected to have a stable, slightly increasing **UER**. **Cluster 1** is supported by sustained resource demand, but it faces ongoing challenges in economic transformation. **Cluster 5** benefits from advantageous policy and governmental investment, but it must guard against a ‘siphon effect’ that diverts resources from surrounding cities. **Cluster 2** (Ecological conservation areas) exhibits the most significant upward trend in **UER**, validating a ‘protection-first’ strategy for ecologically critical zones. The future challenge for this cluster lies in consolidating ecological gains while developing sustainable livelihoods through eco-tourism and eco-agriculture, thereby reducing reliance on external funding.
5. Based on the low-performing indicators and vulnerable cities identified in this study, which serve as potential intervention priorities, a comprehensive monitoring and early-warning system can be constructed to enhance the ecological resilience of cities within the Yellow River Basin. Should the low-performance indicators demonstrate a significant negative trend in vulnerable cities during the specific temporal window (such as the past three years), and the **UER** decline forecasted by the model exceeds the established acceptable threshold, relevant authorities and government entities are advised to undertake proactive interventions aimed at improving the related indicators. Furthermore, in cases where cities from various clusters are involved, it is essential to activate regional coordination mechanisms facilitating joint interventions, thereby ensuring a prompt response to potential ecological resilience issues and risk mitigation.

## Discussions

Classifying 51 YRB prefecture-level cities into seven function-oriented clusters yields more specific, actionable conclusions than administrative zoning (e.g., provinces) in prior literature.

Macro-level zoning assumes homogeneity within regions, thereby obscuring critical differences. For instance, when the basin is divided into Upper, Middle, and Lower Basins, the Upper Basin encompasses both ecologically fragile areas (e.g., some cities in Qinghai) and resource-based cities (e.g., Baiyin, Gansu). The drivers of their **UER** are fundamentally different (policy-driven versus industrial pollution), leading to conclusions that lean toward ‘overall improvement’ and fail to identify the shortcomings of resource-based cities^8,9^. The spatial planning of ecological corridors in the Upper Basin (Qilian Mountain area)^36^ also takes into account major cities such as Xining and Lanzhou, as well as resource cities like Baiyin, within the overall framework of the region. In contrast, this study, by integrating functional clustering with predictive modeling, not only identifies ecologically critical areas but also adds a forward-looking layer to pinpoint future risk hotspots. It indicates that **Cluster 5**—the provincial capital cluster with strong innovation capabilities—and **Cluster 7**—the industrial city cluster in Shandong—can provide technical support to industrial city clusters **3** and **4** in the Upper/Middle Basins. Existing literature, however, can offer only general suggestions, such as strengthening technology diffusion^12^.

For cross-regional planning, this study enables the formulation of more precise and actionable recommendations by revealing the functional connections between non-geographically adjacent clusters. Take ecological compensation, for example: the upper basin (clusters **2**, **4**, and parts of **6**) can act as “suppliers,” providing ecological services such as water-source protection, soil and water preservation, and carbon emission reduction, and receive compensation in the form of financial or technical assistance from the economically developed clusters (7 and 5) in the middle/lower basins that benefit from these services. This necessitates effective basin-wide water governance, including transboundary allocation strategies. Studies on transboundary river basins, such as those employing sequential Rubinstein bargaining models, also provide frameworks for allocating contested water resources among multiple stakeholders^37^. Contrarily, several studies only focused on geographical proximity and planning at the provincial level^38,39^, which may result in suboptimal coordination efficiency, particularly among cities with functionally mismatched roles.

Regarding limitations, the **UER**, derived from objective calculations, identifies statistically influential factors based on correlation, not strict causality. For example, ‘industrial wastewater discharge’ in **Cluster 3** is associated with socio-economic drivers (policies, enforcement) not directly modeled. While SHAP analysis offers valuable insights into feature significance, it predominantly uncovers correlations and associations rather than definitive causality; the identified indicators should be interpreted as potential intervention priorities. Consequently, the translation of these findings into policy necessitates meticulous interpretation informed by domain-specific expertise and potentially supplementary causal inference techniques^40^. Furthermore, while effective, the K-means clustering method introduces subjectivity and oversimplifies complex urban interrelationships. The constructed temporal features can capture short-to-medium dependencies but may miss long-cycle influences like climate trends or the delayed effects of national long-term strategies.

## Conclusions

This study developed and validated an integrated framework combining functional urban clustering with predictive XGBoost modeling to forecast Urban Ecological Resilience (**UER**) in China’s Yellow River Basin. Findings issue critical early warning: continuous **UER** decline in traditional heavy industry cities and fluctuating declines in Loess Plateau and traditional agriculture cities through 2027. Identifying vulnerable cities and low-performing indicators—mainly water stress, industrial pollution, and insufficient environmental investment—enables pre-emptive, targeted interventions beyond retrospective analysis. Results highlight administrative boundary limitations, advocating functional synergy governance like cross-cluster compensation and technology sharing. This framework provides a robust, actionable tool for policymakers to assess future risks and coordinate strategies for the sustainability of the YRB.

Future research could integrate multi-source remote-sensing (e.g., nighttime lights, land-surface temperature), high-resolution stressor data (localized pollution, microclimates), and big data (social-media mobility) for granular **UER** assessments. A dynamic network or fuzzy clustering could capture complex interactions. Advanced deep learning (Transformers, LSTMs) might handle long dependencies with sufficient data. Causal inference methods could elucidate policy-**UER** relationships.

## Supplementary Information

Below is the link to the electronic supplementary material.

Supplementary Material 1

## Data Availability

The data presented in this study are openly available in the China Urban Statistical Yearbook, annually published by China Statistics Press Co., Ltd., from 2010 to 2024. Some of the data are obtained from the Annual Ecological and Environmental Status Bulletins released by provincial governments, and relevant data can also be obtained from the corresponding author upon request.

## References

[1] Alberti, M. & Marzluff, J. M. Ecological resilience in urban ecosystems: linking urban patterns to human and ecological functions. *Urban Ecosyst.* **7**, 241–265. 10.1023/B:UECO.0000044038.90173.c6 (2004).

[2] Stokols, D., Lejano, R. P. & Hipp, J. Enhancing the resilience of human–environment systems: a social ecological perspective. *Ecol. Soc.* **18**, 7. 10.5751/ES-05301-180107 (2013).

[3] Zhang, X. & Li, H. Urban resilience and urban sustainability: what we know and what we do not know? *Cities* **72**, 141–148. 10.1016/j.cities.2017.08.009 (2018).

[4] Dai, W. et al. Analysis of driving forces and dynamic evolution of water ecological security in the Gansu section of the Yellow River Basin. *Arid Zone Res.* **41**, 1662–1671. 10.13866/j.azr.2024.10.05 (2024).

[5] Bai, X. & Shi, P. China’s urbanization at a turning point—challenges and opportunities. *Science* **378**, eabn5759. 10.1126/science.eabn5759 (2025). 10.1126/science.adw3443 40338998

[6] Zhang, J. et al. Multi-scale effects of meteorological conditions and anthropogenic emissions on PM2.5 concentrations over major cities of the Yellow River Basin. *Int. J. Environ. Res. Public. Health*. **19**, 15060. 10.3390/ijerph192215060 (2022). 36429779 10.3390/ijerph192215060 PMC9690158

[7] Wang, Z., Fu, H. & Ren, X. Assessing the effects of extreme climate risk on urban ecological resilience in China. *Environ. Sci. Pollut Res.* **31**, 28225–28240. 10.1007/s11356-024-33039-w (2024). 10.1007/s11356-024-33039-w 38536570

[8] Li, C. et al. Spatiotemporal dynamics and spatial correlation patterns of urban ecological resilience across the Yellow River Basin in China. *Sci. Rep.* **14**, 31286. 10.1038/s41598-024-82675-2 (2024). 39732787 10.1038/s41598-024-82675-2 PMC11682375

[9] Lu, F., Liu, Q. & Wang, P. Spatiotemporal characteristics of ecological resilience and its influencing factors in the Yellow River Basin of China. *Sci. Rep.* **14**, 16988. 10.1038/s41598-024-67628-z (2024). 39043742 10.1038/s41598-024-67628-z PMC11266585

[10] Miao, C. et al. Urban resilience evaluation based on entropy-TOPSIS model: a case study of county-level cities in Ningxia, Northwest China. *Int. J. Environ. Sci. Technol.* **22**, 4187–4202. 10.1007/s13762-024-05880-6 (2025).

[11] Dong, Y., Cai, J., Dong, W. W., Wang, N. & An, Q. The impact of Urban residential areas on bird diversity: A case study from Harbin, Northeast China. *Habitat Int.* **163**, 103463. 10.1016/j.habitatint.2025.103463 (2025).

[12] Huang, J. et al. Spatial-temporal differentiation and driving factors of ecological resilience in the Yellow River Basin, China. *Ecol. Indic.* **154**, 110763. 10.1016/j.ecolind.2023.110763 (2023).

[13] Chen, H. et al. Evaluating the impact of urbanization on ecological resilience in the Yellow River Basin using remote sensing data. *Sustainability* **17**, 7114. 10.3390/su17157114 (2025).

[14] Liu, C. et al. Revealing spatiotemporal interaction patterns behind complex cities. *Chaos* **32**, 081105. 10.1063/5.0098132 (2022). 36049958 10.1063/5.0098132

[15] Kartal, V. et al. Prediction of groundwater drought based on hydro-meteorological insights via machine learning approaches. *Phys. Chem. Earth A/B/C*. **136**, 103757. 10.1016/j.pce.2024.103757 (2024).

[16] Alkanjo, S. et al. Machine Learning as a Tool to Predict Reference Evapotranspiration. *Water Resour. Manage.* **40**, 97. 10.1007/s11269-025-04460-8 (2026).

[17] Ran, X. et al. Application of explainable machine learning in the study of urban ecological economic resilience. *Eco Model.* **511**, 111368. 10.1016/j.ecolmodel.2025.111368 (2026).

[18] Yang, Z. et al. Spatio-temporal heterogeneity and influencing factors in the synergistic enhancement of urban ecological resilience: evidence from the Yellow River Basin of China. *Appl. Geogr.* **173**, 103459. 10.1016/j.apgeog.2024.103459 (2024).

[19] Wang, J. et al. Machine learning approaches for predicting urban ecological resilience under climate change scenarios. *Sci. Rep.* **14**, 7364. 10.1038/s41598-024-67364-4 (2024).38548903

[20] Krasny, M. E., Lundholm, C. & Plummer, R. Resilience in social–ecological systems: the roles of learning and education. *Environ. Educ. Res.* **16**, 463–474. 10.1080/13504622.2010.505416 (2010).

[21] Fang, A. M. et al. Trade-off and synergy relationships and regional regulation of multifunctional cultivated land in the Yellow River Basin. *Front. Environ. Sci.* **13**, 1542002. 10.3389/fenvs.2025.1542002 (2025).

[22] Zhang, Q. et al. Comprehensive climate-ecological-hydrological zoning of the Yellow River Basin using K-means clustering. *Water Sav. Irrig.* **7**, 59–65. 10.12396/jsgg.2024487 (2025).

[23] Li, X., Wang, Y. & Zhang, L. Dynamic assessment of ecological resilience and its driving factors in the Yangtze River Basin. *Reg. Sustain.* **5**, 100159. 10.1016/j.regsus.2024.100159 (2024).

[24] Liu, Y. et al. Impacts of land use changes on water ecological security in the Yellow River Basin. *Sci. Total Environ.* **904**, 162580. 10.1016/j.ecolind.2024.112212 (2023).

[25] An, Q., Dong, Y., Dong, W. & Xiao, S. Spatiotemporal dynamics and nonlinear landscape-driven mechanisms of urban heat islands in a winter city: A case study of Harbin, China. *Sustain. Cities Soc.* **133**, 106842. 10.1016/j.scs.2025.106842 (2025).

[26] Ma, T. et al. An interpretable hybrid model for predicting step-like landslide displacement: a case study in the Three Gorges Reservoir. *Nat. Hazards*. **121**, 21441–21458. 10.1007/s11069-025-07638-w (2025).

[27] Chen, T. & Guestrin, C. XGBoost: a scalable tree boosting system. *Proc. 22nd ACM SIGKDD Int. Conf. Knowl. Discov Data Min.* **785-794**10.1145/2939672.2939785 (2016).

[28] Kumar, R. et al. Modeling and forecasting rainfall patterns in India: a time series analysis with XGBoost algorithm. *Environ. Earth Sci.* **83**, 256. 10.1007/s12665-024-11576-4 (2024).

[29] Lundberg, S. M. & Lee, S. I. A unified approach to interpreting model predictions. *Adv. Neural Inf. Process. Syst.* **30**, 4765–4774. 10.5555/3295222.3295230 (2017).

[30] Wang, Z. Validation, robustness, and accuracy of perturbation-based sensitivity analysis methods for time-series deep learning models. *Proc. AAAI Conf. Artif. Intell.* **38** (23768-23770). 10.1609/aaai.v38i22.30717 (2024).

[31] Lai, M. et al. Temporal cross-validation in forecasting: a case study of COVID-19 incidence using wastewater data. *Qual. Reliab. Eng. Int.* **41**, 672–688. 10.1002/qre.3742 (2025).

[32] Setiyorini, T. & Frieyadie, F. Comparison of the application of linear regression with sliding window validation and K-fold cross-validation for forecasting Covid-19 recovered cases. *J. Ris Inf.* **6**, 159–166. 10.34288/jri.v6i2.345 (2024).

[33] Shortridge, J. E. & Guikema, S. D. Scenario discovery with multiple criteria: an evaluation of the robust decision-making framework for climate change adaptation. *Risk Anal.* **36**, 2298–2312. 10.1111/risa.12505 (2016). 26890212 10.1111/risa.12582

[34] Yuan, Z. & Hu, W. Urban resilience to socioeconomic disruptions during the COVID-19 pandemic: evidence from China. *Int. J. Disaster Risk Reduct.* **91**, 103670. 10.1016/j.ijdrr.2023.103670 (2023). 37041883 10.1016/j.ijdrr.2023.103670 PMC10073087

[35] Zhang, Q. et al. Impact of the emergency response to COVID-19 on air quality and its policy implications: evidence from 290 cities in China. *Environ. Sci. Policy*. **145**, 10–23. 10.1016/j.envsci.2023.103670 (2023). 10.1016/j.envsci.2023.04.009 PMC10093300 37070073

[36] Yang, L. et al. A Refined Framework for Alpine Ecological Security Patterns: Identifying Key Restoration Areas under Different Scenarios. *Ecosyst. Health Sustain.* **12**, 0465. 10.34133/ehs.0465 (2026).

[37] Yuan, L. et al. Allocating water resources in transboundary river basins: a sequential Rubinstein bargaining approach with risk discounting. *J. Hydrol. Reg. Stud.* **63**, 102989. 10.1016/j.ejrh.2025.102989 (2026).

[38] Lu, H. & Zhao, X. Investigating the horizontal carbon ecological compensation mechanism in the Yellow River Basin: construction, validation, and policy impact. *Front. Environ. Sci.* **13**, 1511882. 10.3389/fenvs.2025.1511882 (2025).

[39] Hu, H., Tian, G., Wu, Z. & Xia, Q. Cross-regional ecological compensation under the composite index of water quality and quantity: A case study of the Yellow River Basin. *Environ. Res.* **239**, 117152. 10.1016/j.envres.2023.117152 (2023). 10.1016/j.envres.2023.117152 37717804

[40] Dillon, E., LaRiviere, J., Lundberg, S., Roth, J. & Syrgkanis, V. Accessed March 01, Be careful when interpreting predictive models in search of causal insights. SHAP documentation. (2026). https://shap.readthedocs.io/en/latest/example_notebooks/overviews/Be%20careful%20when%20interpreting%20predictive%20models%20in%20search%20of%20causal%20insights.html

