# Supplementary material for: Predicting the future of urban ecological resilience in China’s Yellow River Basin: a machine learning approach
Source: Sci Rep. 2026 May 24;16:23763. doi: 10.1038/s41598-026-54737-0 (PMC13429617; doi:10.1038/s41598-026-54737-0)
Supplement: Supplementary file 1 — Supplementary Material 1 [file 41598_2026_54737_MOESM1_ESM.docx]

Predicting the future of urban ecological resilience in China’s Yellow River Basin: A machine learning approach

Ting Fan^1*^, Xiaoyong Li^2^, Chenlu Huang^1^, Guan Huang^1^

^1^Institute of Human Geography, College of Tourism, Xi’an International Studies University, Xi’an, 710128, People’s Republic of China；

^2^School of Tourism and Culinary, Yangzhou University, Yangzhou, 225009, People’s Republic of China；

*Corresponding author: [fanting@xisu.edu.cn](mailto:fanting@xisu.edu.cn);

**1. Indicators**

**Supplemental Table 1:** Statistical description of the raw data of indicators

| **Indicators** | **Min** | **Max** | **Mean** | **Std** | **Data Loss%*** |
| --- | --- | --- | --- | --- | --- |
| N1 Urban High-temperature Days (days/a) | 3 | 90 | 55 | 10.2 |  |
| N2 Water Resource per Capita (m^3^) ** | 50 | 950 | 473 | 132 |  |
| N3 Extreme Weather (days/a)*** | 15 | 120 | 36 | 15.3 | 1.1 |
| N4 Urban Air Quality (days/a） | 120 | 320 | 160 | 13.5 |  |
| N5 Desertified Land Ratio (%) | 1.5 | 53.6 | 5.8 | 7.2 |  |
| N6 Forest Coverage Rate (%) | 3.2 | 70.5 | 38.3 | 10.3 |  |
| E1 GDP per capita (thousand, CNY/a) | 32 | 162 | 78 | 20.5 |  |
| E2 Second Industrial Added Value (billion CNY/a) | ​​3.5 | 320.2 | 46.3 | 55.6 |  |
| E3 Ratio of Education and Research Budget to GDP (%) | ​​0.3 | 10.2 | 2.1 | 2.45 | 2.0 |
| E4 Ratio of Environmental Protection Budget to GDP (%) | 1.1 | 3.5 | 1.6 | 0.5 |  |
| U1 Population Density (per km^2^) | 35 | 13258 | 5300 | 63.6 |  |
| U2 Industrial Wastewater Discharge (tons per million GDP) | 5.4 | 185.3 | 12.8 | 23.5 | 0.5 |
| U3 Utilization rate of Industrial Solid Waste (%) | 85 | 99 | 89 | 1.1 | 1.2 |
| U4 Urbanization Rate (%) | 38 | 81 | 61 | 5.2 |  |
| U5 Urban Green Coverage (%) | 35 | 46 | 40 | 1.2 |  |

* For lost data, imputation was applied using the mean data of the same cluster in the same year to prevent information leakage across time.

** **N2**: The total available renewable water resources (surface water and groundwater) within a defined administrative area divided by its permanent population, in the China Urban Statistical Yearbook.

*****N3**: Referring to Measures for the Release and Dissemination of Meteorological Disaster Alert Signals of China

(1) Heavy rain: The number of days when a red alert is issued, meaning the rainfall within 3 hours will reach over 100 millimeters, or has already reached over 100 millimeters, and the rainfall may continue.

(2) Strong wind: The number of days when an orange alert is issued, meaning that within 6 hours of that day, there may be a strong wind affecting the area, with an average wind force of over 10, or gusts of over 11; or there has been an average wind force of 10-11 and gusts of 11-12, and it may continue.

(3) Hail: Days of hail, based on Annual Climate Bulletin.

**Legend:**  This table provides a statistical description of the raw data for the 15 indicators used in assessing Urban Ecological Resilience (**UER**) in the Yellow River Basin between 2010 and 2024. For each indicator, it lists the minimum, maximum, mean, and standard deviation observed across the dataset. It also notes the percentage of data loss for specific indicators (N3, E3, U2, U3). The indicators are categorized into Natural Conditions (N1-N6), Economic Conditions (E1-E4), and Urban Management (U1-U5), covering aspects like climate extremes, water resources, air quality, land use, economic development, education investment, environmental protection spending, population density, pollution discharge, waste utilization, urbanization rate, and green coverage. This table details the range and variability of the input data, with a note on data imputation for missing values.

**2. K-means classification of city clusters**

**Supplemental Table 2:** The Classification of City Clusters in the Yellow River Basin based on ecological background and industries. (The geographical location of cities can be found in Supplementary Figure 2 through the four-digit administrative code in parentheses)

| **City Cluster** | **Cities  (Administrative**  **Code)** | **Character and its encoded/value used in the K-means Classifier** | | | |
| --- | --- | --- | --- | --- | --- |
|  |  | **Land**  **Use Type** | **​​Land form** | **​​The largest industry and its ratio to the total (mean)** | **Ratio of the Added Value of the Third Industry/Second**  **Industry(mean)** |
| 1  Resource-Based Industry | Baiyin  Yulin  Ordos  Xinzhou  Jincheng  Dongying | Bare land  Cropland | Plain  Mountainous terrain | Second Industry  0.72 | 0.42 |
| 2  Ecological Conservation | Yushu  Guoluo  Huangnan  Haidong  Haibei  Hainan Gannan  Linxia  Dingxi | Grassland  Bare land | Mountainous terrain  Tibet Plateau  Loess Plateau | First Industry  0.61 | 0.65 |
| 3  Traditional  Heavy Industry | Shizuishan(6402)  Yinchuan  Linfen(1410) Huhehot  Taiyuan  Baoji  Baotou(1502)  Luoyang  Jiaozuo | Cropland | Plai  Mountainous terrain | Second Industry  0.67 | 0.22 |
| 4  Loess Plateau  Agriculture | Tianshui  Pingliang(6208)  Guyuan(6404)  Qingyang (6210)  Wuzhong  Yan’an  Lvliang | Cropland  Bare Land | Loess Plateau | First Industry  0.72 | 0.35 |
| 5  Regional Centre | Lanzhou  Xi’an  Zhengzhou  Jinan | Built-up area  of urban | Plain | Third Industry  0.46 | 1.26 |
| 6  Traditional Agriculture | Zhongwei(6405)  Wuhai  Bayan Nur  Xianyang  Weinan  Heze(3717)  Yuncheng(1408)  Sanmenxia  Kaifeng  Xinxiang  Puyang | Cropland | Plain  Mountainous terrain | First Industry  0.62 | 0.52 |
| 7  Industry and Agriculture | Liaocheng  Dezhou  Binzhou  Zibo  Tai’an | Cropland  Built-up area  of urban | Plain | Second Industry  0.51 | 1.05 |

**Legend:** This table outlines the classification of 51 prefecture-level cities within the Yellow River Basin into seven distinct functional city clusters, based on their ecological background and industrial characteristics. For each cluster, it lists representative cities with their administrative codes. It then describes the key features used in the K-means classification for each cluster: predominant Land Use Type (e.g., Bare land, Cropland), Land Form (e.g., Plain, Mountainous terrain), the largest industry and its mean ratio to total economic activity, and the mean ratio of the added value of the Third Industry to the Second Industry. This classification aims to group cities with similar ecological conditions and industrial structures, enabling more targeted policy analysis and recommendations.

**Supplemental Table 3** Feature vector of categorical variable in K-means Classification of city Cluster

| **Land Use Type** | **Vector** | **Land Form** | **Vector** | **Industry** | **Vector** |
| --- | --- | --- | --- | --- | --- |
| Barren land | 1 0 0 0 | Plain | 1 0 0 0 | First Industry | 1 0 0 |
| Crop land | 0 1 0 0 | Loess Plateau | 0 1 0 0 | Second Industry | 0 1 0 |
| Built-up area of urban | 0 0 1 0 | Mountainous terrain | 0 0 1 0 | Third Industry | 0 0 1 |
| Grassland | 0 0 0 1 | Tibet Plateau | 0 0 0 1 | - | - |

**Legend:** This table defines the vector representations for categorical variables used in the K-means classification of city clusters. It specifies how each category within 'Land Use Type,' 'Land Form,' and 'Industry' is encoded into a numerical vector. For instance, 'Barren land' is represented as '1 0 0 0', 'Cropland' as '0 1 0 0', and so on for land use. Similarly, different landforms and industrial types are assigned unique vector representations. This standardized encoding allows for the quantitative input of categorical data into the K-means clustering algorithm, facilitating the identification of functional similarities among cities.

**Supplemental Table 4:** The Silhouette value of cluster number and cluster in the K-means method

| **Number of Clusters** | **Silhouette** | **Stability Index** | **Cluster Size Consistency** | **Cluster** | **Mean Silhouette** |
| --- | --- | --- | --- | --- | --- |
| 5 | 0.592 | 0.6328 | 0.3928 | **1** | 0.865 |
| 6 | 0.781 | 0.6546 | 0.1624 | **2** | 0.851 |
| 7 | 0.823 | 0.7142 | 0.1899 | **3** | 0.789 |
| 8 | 0.799 | 0.6072 | 0.1882 | **4** | 0.775 |
| 9 | 0.752 | 0.6743 | 0.1727 | **5** | 0.832 |
| 10 | 0.656 | 0.7065 | 0.3099 | **6** | 0.716 |
| 11 | 0.501 | 0.7019 | 0.4047 | **7** | 0.775 |

**Legend:** This table presents the results of the K-means clustering analysis, evaluating the optimal number of clusters for categorizing cities in the Yellow River Basin. It shows the Silhouette value, Stability Index, and Cluster Size Consistency for clustering solutions ranging from 5 to 11 clusters. The Silhouette value measures how well each city is matched to its own cluster compared to other clusters, with higher values indicating better clustering. The Stability Index assesses the robustness of the clustering across multiple random initializations. The table indicates that K=7 achieved the highest Silhouette value (0.823) and a good Stability Index (0.7142), suggesting it is the most appropriate number of clusters for grouping the cities into functionally similar groups.

**Supplemental Figure 1:** The geographical location of cities mentioned in Table 4 and Supplementary Table 2 with the numerical code

**
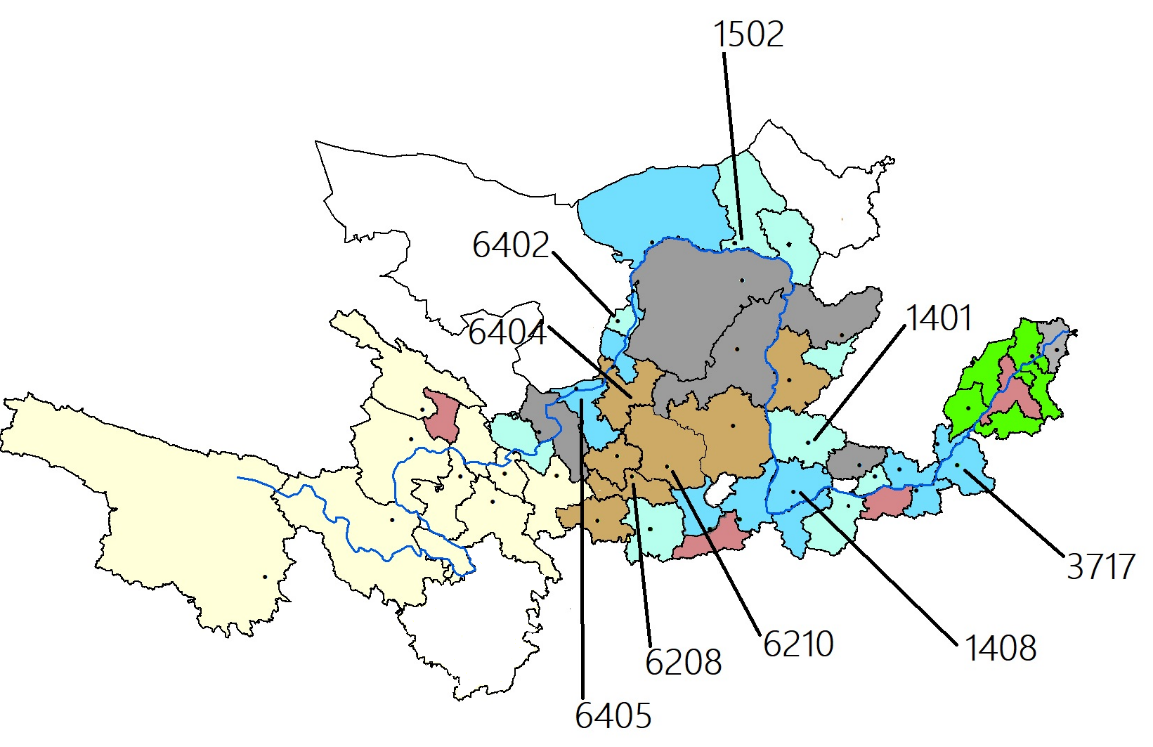
**

**Legend:** This figure displays a map illustrating the geographical locations of cities mentioned in Table 4 and Supplemental Table 2. Each city is marked with a numerical code, which corresponds to the four-digit administrative code provided in parentheses within those tables. This visual representation allows readers to locate the specific cities belonging to each cluster and understand their spatial distribution within the Yellow River Basin, complementing the tabular data by providing a geographic context for the functional clustering.

**3. Model:** Temporal construction of samples and hyperparameters

**Supplemental Table 5:** The temporal construction of samples for three independent XGBoost models

|  | **Group** | **Period of input viable** | **Year of Target Output Viable** | **Total Samples** |
| --- | --- | --- | --- | --- |
| Model **M1** | Training | 2010-2012 | 2013 | 49 |
|  |  | 2011-2013 | 2014 |  |
|  |  | 2012-2014 | 2015 |  |
|  |  | 2013-2015 | 2016 |  |
|  |  | 2014-2016 | 2017 |  |
|  |  | 2015-2017 | 2018 |  |
|  |  | 2016-2018 | 2019 |  |
|  | Validation | 2017-2019 | 2020 | 21 |
|  |  | 2018-2020 | 2021 |  |
|  |  | 2019-2021 | 2022 |  |
|  | Test | 2020-2022 | 2023 | 14 |
|  |  | 2021-2023 | 2024 |  |
|  | Prediction | 2022-2024 | 2025 | 7 |
| Model **M2** | Training | 2010-2012 | 2014 | 42 |
|  |  | 2011-2013 | 2015 |  |
|  |  | 2012-2014 | 2016 |  |
|  |  | 2013-2015 | 2017 |  |
|  |  | 2014-2016 | 2018 |  |
|  |  | 2015-2017 | 2019 |  |
|  | Validation | 2016-2018 | 2020 | 21 |
|  |  | 2017-2019 | 2021 |  |
|  |  | 2018-2020 | 2022 |  |
|  | Test | 2019-2021 | 2023 | 14 |
|  |  | 2020-2022 | 2024 |  |
|  | Prediction | 2022-2024 | 2026 | 7 |
| Model **M3** | Training | 2010-2012 | 2015 | 35 |
|  |  | 2011-2013 | 2016 |  |
|  |  | 2012-2014 | 2017 |  |
|  |  | 2013-2015 | 2018 |  |
|  |  | 2014-2016 | 2019 |  |
|  | Validation | 2015-2017 | 2020 | 21 |
|  |  | 2016-2018 | 2021 |  |
|  |  | 2017-2019 | 2022 |  |
|  | Test | 2018-2020 | 2023 | 14 |
|  |  | 2019-2021 | 2024 |  |
|  | Prediction | 2022-2024 | 2027 | 7 |

**Legend:** This table details the temporal construction of samples for three independent XGBoost models (**M1**, **M2**, **M3**) used for predicting Urban Ecological Resilience (**UER**). It specifies the range of years used for input variables (historical **UER** and engineered temporal features) and the target output variable (future **UER**) for each model's training, validation, test, and prediction phases. For example, Model **M1** uses data from 2010-2012 to predict 2013, and its prediction phase covers 2022-2024 to forecast **UER** for 2025. This structured approach ensures that the models are trained and tested on appropriate time windows, enabling accurate forecasting for future years.

**Supplemental Table 6:** The hyperparameter for XGBoost models （**M1**, **M2**, **M3**）

| **Hyperparameter** | **Value** |
| --- | --- |
| objective | reg:squarederror |
| booster | gbtree |
| tree_method | hist |
| max_depth | 4 |
| learning_rate | 0.05 |
| n_estimators | 300 |
| early_stopping_rounds | 100 |
| subsample | 0.8 |
| colsample_bytree | 0.8 |
| gamma | 0.05 |
| reg_alpha | 0 |
| reg_lambda | 1 |
| min_child_weight | 1 |
| random_state | 42 |

**Legend:** This table lists the key hyperparameters and their chosen values for the XGBoost models (**M1**, **M2**, **M3**) used in this study. These parameters control the learning process and the structure of the decision trees, influencing model performance and preventing overfitting. Important parameters include objective (the loss function), booster type (gbtree), max_depth (tree complexity), learning_rate (step size shrinkage), n_estimators (number of trees), subsample (fraction of samples used per tree), and colsample_bytree (fraction of features used per tree). These settings were optimized for effective **UER** prediction.

**Supplemental Table 7:** The temporal construction of samples in cross-validation of XGBoost Models

|  | **Training Group** | **Test Group** | **Total Samples** |
| --- | --- | --- | --- |
| Model **M1** | 2010-2015 | 2016 | 42 |
|  | 2010-2016 | 2017 |  |
|  | 2010-2017 | 2018 |  |
|  | 2010-2018 | 2019 |  |
|  | 2010-2019 | 2020 |  |
|  | 2010-2020 | 2021 |  |
| Model **M2** | 2010-2015 | 2017 | 35 |
|  | 2010-2016 | 2018 |  |
|  | 2010-2017 | 2019 |  |
|  | 2010-2018 | 2020 |  |
|  | 2010-2019 | 2021 |  |
| Model **M3** | 2010-2015 | 2018 | 28 |
|  | 2010-2016 | 2019 |  |
|  | 2010-2017 | 2020 |  |
|  | 2010-2018 | 2021 |  |

**Legend:** This table illustrates the temporal construction of samples used for cross-validation (**CV**) of the XGBoost models (**M1**, **M2**, **M3**). It shows how the dataset is chronologically partitioned into training and testing groups. For example, Model **M1** uses data from 2010-2015 for training to predict 2016, then rolls forward to train on 2010-2016 to predict 2017, and so on. This time-series cross-validation approach ensures that the model is evaluated on unseen future data at each step, providing a robust assessment of its generalization capability and stability.

**Supplemental Table 8**. The sample constitution in the simulation test with and without policy intervention by improving or deteriorating the low-performing indicators of vulnerable cities in clusters **3**/**4**/**6**.

| **Year** | **Intervention Condition** | **Comparison (without Intervention)** |
| --- | --- | --- |
| 2024 | Original raw values of indicators | Original raw values of indicators |
| 2025 | Indicators’ values with 10% improvement based on 2024 | Indicators’ values with 5% deterioration based on 2024 |
| 2026 | Indicators’ values with 10% improvement based on 2025 | Indicators’ values with 5% deterioration based on 2025 |
| 2027 | **UER** Predicted by **M1** using updated indicators’ values of 2024-2026 | **UER** Predicted by **M1** using updated indicators’ values of 2024-2026 |
| 2028 | **UER** Predicted by **M2** using updated indicators’ values of 2024-2026 | **UER** Predicted by **M2** using updated indicators’ values of 2024-2026 |
| 2029 | **UER** Predicted by **M3** using updated indicators’ values of 2024-2026 | **UER** Predicted by **M3** using updated indicators’ values of 2024-2026 |

Legend: This table outlines the sample constitution for the simulation tests, comparing scenarios with and without policy intervention aimed at improving low-performing indicators in vulnerable cities within clusters **3**, **4**, and **6**. It details the indicator values for 2024 as the baseline. For 2025 and 2026, it shows that the "Intervention Condition" involves a 10% annual improvement in positive indicators and a corresponding decrease in negative indicators, while the "Comparison (without Intervention)" scenario shows a 5% annual deterioration. The **UER** for 2027–2029 is then predicted using these updated indicator values for each scenario, allowing for a quantitative evaluation of intervention effectiveness.

**4. Example of an explanation of Models**

**Supplemental Table 9**. Explanation of Model-Prediction results for **Cluster 3** (Traditional Heavy Industry), and the identification of vulnerable cities and indicators

| **Features**  **City** | **Important Temporal Features (SHAP value)** | **Diff_mean_**  **(City-level)** | **Roll_mean_**  **(City-level)** | **High-weight indicators**  **(value)** | **Low-performing Indicators**  **with minimum *w*×*r* (**  **Equation 6)** |
| --- | --- | --- | --- | --- | --- |
| Yinchuan | Diff_mean_(0.45)  Roll_mean_(0.38)  Roll_std_ (0.09)  Lag_3(0.05)  Diff_2(0.02) | -0.002 | 0.61 | **E3** 0.115  **E2** 0.105  **N2** 0.084  **N5** 0.078  **U2**  0.076  **N4** 0.067  **E4** 0.075 | - |
| Shizuishan |  | -0.011 | 0.49 |  | **E4**(0.009) **N4**(0.013)  **N2**(0.016) |
| Baotou |  | -0.006 | 0.53 |  | **N4**(0.011);**U2**(0.012);**E2**(0.023), |
| Huhehot |  | 0.001 | 0.65 |  | - |
| Taiyuan |  | -0.002 | 0.64 |  | - |
| Baoji |  | -0.001 | 0.68 |  | - |
| Linfen |  | -0.013 | 0.51 |  | **U2**(0.008);**N2**(0.012), **N4**(0.013);**E2** (0.013) |
| Luoyang |  | 0.002 | 0.66 |  | - |
| Jiaozuo |  | -0.004 | 0.54 |  | - |

Legend: This table details the explanation of model prediction results for **Cluster 3** (Traditional Heavy Industry). It identifies important temporal features (like Diff_mean_ and Roll_mean_) that significantly influence the predicted **UER** decline, based on SHAP values at the city level. It then lists the vulnerable cities within this cluster (e.g., Yinchuan, Shizuishan, Baotou, Linfen) that their hostorical ***uer*** contribute most to the decline of important temporal features (Diff_mean_ and Roll_mean_ ). For each vulnerable city, it highlights "High-weight indicators" based on their cumulative weights from EWM and then specifically identifies "Low-performing Indicators" that have a minimum weighted product (w×r) and often show decreasing trends, representing key targets for intervention.

**Supplemental Table 10.** The selection of high-weight indicators and low-performing indicators from vulnerable cities in **Cluster 3** (red text indicates selected indicators in process, with increasing weight ***w***_j_ and decreasing ***w***_j_×***r***_ij_)

|  | ***w***_j_ | | | ***w***_j_×***r***_ij_ | | | | | | | | |
| --- | --- | --- | --- | --- | --- | --- | --- | --- | --- | --- | --- | --- |
|  |  |  |  | Shizuishan, ***uer***_mean_0.52 | | | Linfen, ***uer***_mean_ 0.49 | | | Baotou, ***uer***_mean_0.59 | | |
|  | 2022 | 2023 | 2024 | 2022 | 2023 | 2024 | 2022 | 2023 | 2024 | 2022 | 2023 | 2024 |
| N1 | 0.066 | 0.060 | 0.058 |  |  |  |  |  |  |  |  |  |
| N2 | 0.08 | 0.085 | 0.087 | 0.022 | 0.015 | 0.013 | 0.016 | 0.013 | 0.009 | 0.046 | 0.043 | 0.049 |
| N3 | 0.068 | 0.065 | 0.062 |  |  |  |  |  |  |  |  |  |
| N4 | 0.064 | 0.067 | 0.070 | 0.015 | 0.013 | 0.013 | 0.014 | 0.010 | 0.010 | 0.012 | 0.013 | 0.010 |
| N5 | 0.076 | 0.078 | 0.080 | 0.035 | 0.032 | 0.034 | 0.036 | 0.037 | 0.039 | 0.046 | 0.045 | 0.048 |
| N6 | 0.063 | 0.056 | 0.054 |  |  |  |  |  |  |  |  |  |
| E1 | 0.065 | 0.065 | 0.064 |  |  |  |  |  |  |  |  |  |
| E2 | 0.100 | 0.106 | 0.110 | 0.050 | 0.059 | 0.061 | 0.015 | 0.014 | 0.010 | 0.025 | 0.024 | 0.020 |
| E3 | 0.113 | 0.116 | 0.117 | 0.036 | 0.035 | 0.036 | 0.039 | 0.038 | 0.041 | 0.046 | 0.048 | 0.047 |
| E4 | 0.074 | 0.076 | 0.077 | 0.011 | 0.009 | 0.007 | 0.045 | 0.043 | 0.044 | 0.051 | 0.053 | 0.052 |
| U1 | 0.012 | 0.011 | 0.012 |  |  |  |  |  |  |  |  |  |
| U2 | 0.073 | 0.075 | 0.078 | 0.055 | 0.050 | 0.052 | 0.009 | 0.006 | 0.008 | 0.015 | 0.009 | 0.010 |
| U3 | 0.048 | 0.049 | 0.047 |  |  |  |  |  |  |  |  |  |
| U4 | 0.065 | 0.060 | 0.053 |  |  |  |  |  |  |  |  |  |
| U5 | 0.033 | 0.031 | 0.031 |  |  |  |  |  |  |  |  |  |

Legend: This table details the selection process for high-weight and low-performing indicators for vulnerable cities in **Cluster 3** (Traditional Heavy Industry) during 2022-2024, specifically for Shizuishan, Linfen, and Baotou. It shows the indicator weights (***w***_j_) and the product of weight and normalized value (***w***_j_×***r***_ij_) for various indicators across the three years. Indicators are first selected based on a cumulative weight sum exceeding 0.6 (high-weight indicators). Among these, those with a decreasing ***w***_j_×***r***_ij_ trend and contributing less than 5% of the total are identified as low-performing indicators. Red text highlights indicators selected during the process, illustrating the methodology for pinpointing specific issues driving **UER** decline.

**5. Program and Codes**

**Supplemental Figure 2:** The flowchart of the typical practice of classification, model training, prediction, and comparison by ***Orange 3***. (Model M1 as an example)


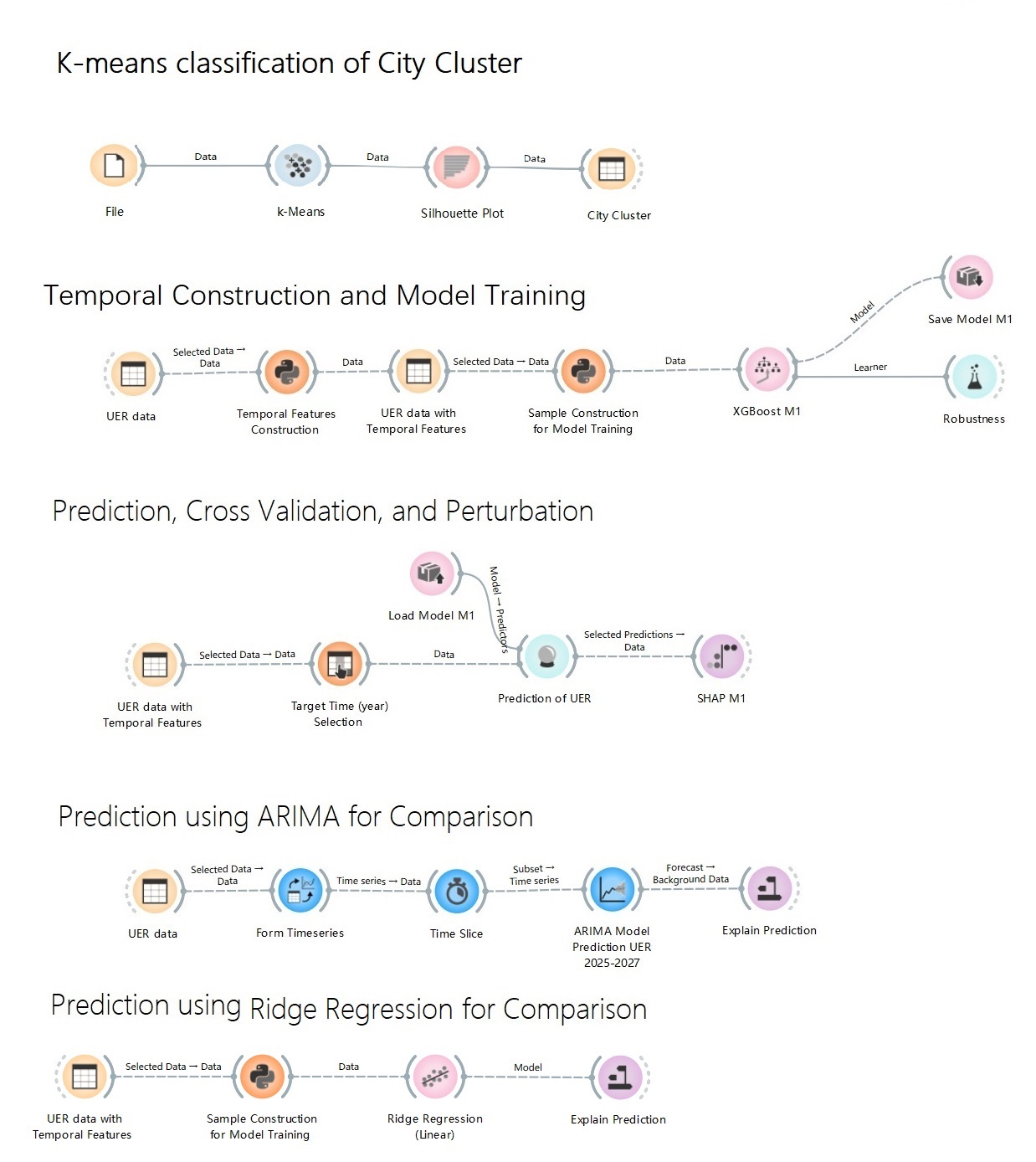


Legend: This figure presents a flowchart illustrating the typical workflow implemented using Orange 3 software for classification, model training, prediction, and comparison, using Model **M1** as an example. The flowchart shows sequential steps, including data input, applying K-means clustering to form city clusters, preparing temporal features for each cluster, constructing samples for model training and prediction, training the XGBoost model, generating predictions, and comparing model performance. This visual guide demonstrates how the different components of the research methodology are integrated and executed within the Orange 3 environment. The comparison modules (ARIMA, Ridge) are applied analogously for all prediction models.

**Supplemental Text 1**. Selected critical codes for ‘temporal features construction’ widget in **Supplementary Figure 2** (Cluster 3 and Model M1 as example)

Legend: This text provides critical Python code snippets for the 'temporal features construction' widget within the Orange 3 workflow (as shown in Supplemental Figure 2), using Cluster 3 and Model M1 as an example. The code demonstrates how to read raw UER data, calculate various temporal features including lag effects (Lag_1, Lag_2, Lag_3), rolling statistics (Rollmean, Rollstd), and difference features (Diff_1, Diff_2, Diffmean). These features are then appended to the original data, creating a new CSV file containing the UER time series enriched with temporal information, which is essential for the subsequent XGBoost modeling.

import numpy as np

import pandas as pd

# Parameters and paths

input_csv_path = "Cluster_3_2010_2024_with_temporaLfeatures.csv" # Original data

city_col = "City"

year_start, year_end = 2010, 2024

# Read base data and prepare year columns

df = pd.read_csv(input_csv_path)

year_cols = [str(y) for y in range(year_start, year_end + 1)]

df = df[[city_col] + year_cols].sort_values(city_col).reset_index(drop=True)

uer = df[year_cols].astype(float)

# 1) Lag features

def build_lag_features(u, lags=[1, 2, 3]):

return {f"Lag_{lag}": u.shift(lag, axis=1) for lag in lags}

# 2) Rolling mean and std based on Lag_1

def build_roll_features(lag1):

roll_mean = lag1.rolling(window=3, min_periods=1, axis=1).mean()

roll_std = lag1.rolling(window=3, min_periods=1, axis=1).std(ddof=0)

return roll_mean, roll_std

# 3) Difference features

def build_diff_features(u, lags=[1, 2]):

diffs = {f"Diff_{lag}": (u - u.shift(lag, axis=1)) for lag in lags}

diff_mean = sum(diffs.values()) / len(lags)

return diffs, diff_mean

# Build temporal features

lag_feats = build_lag_features(uer)

Lag_1 = lag_feats["Lag_1"]

Rollmean, Rollstd = build_roll_features(Lag_1)

Diffs, Diffmean = build_diff_features(uer)

#Helper: rename columns to wide feature names

def to_wide(df_like, name_prefix):

out = df_like.copy()

out.columns = [f"{name_prefix}_{c}" for c in out.columns]

return out

# Collect feature frames concisely

temporal_frames = [

to_wide(Lag_1, "Lag_1"),

to_wide(lag_feats["Lag_2"], "Lag_2"),

to_wide(lag_feats["Lag_3"], "Lag_3"),

to_wide(Rollmean, "Rollmean"),

to_wide(Rollstd, "Rollstd"),

to_wide(Diffs["Diff_1"], "Diff_1"),

to_wide(Diffs["Diff_2"], "Diff_2"),

to_wide(Diffmean, "Diffmean")

]

# Concatenate all features to the original data

out_with_features = pd.concat([df] + temporal_frames, axis=1)

# Output CSV

output_csv_path = "Cluster_3_2010_2024_with_temporal_features_FIXED.csv"

out_with_features.to_csv(output_csv_path, index=False)

print("Temporal features constructed and saved to:", output_csv_path)

print("Shape sanity check - Original df:", df.shape, "Output df:", out_with_features.shape)

**Supplementary Text 2**. Selected critical codes for widget ‘sample construction for model training’ in **Supplementary Fig.2** (Cluster 3 and Model M1 as example)

Legend: This text provides critical Python code snippets for the 'sample construction for model training' widget within the Orange 3 workflow (as shown in Supplemental Figure 2), using **Cluster 3** and Model **M1** as an example. The code defines the training, validation, and test sets based on specific target years. It iterates through each target year, constructs input samples using a sliding window of historical data and engineered temporal features, and pairs them with the corresponding target **UER**. The code also handles data filtering for valid samples and categorizes them according to the defined temporal splits, preparing the data in the format required by the XGBoost model.

import numpy as np

import pandas as pd

# Parameters and paths

city_col = "City"

year_start, year_end = 2010, 2024

window_size = 3

input_csv_path = "Cluster_3_2010_2024_with_temporal_features_FIXED.csv"

# Read data with features

df_feat = pd.read_csv(input_csv_path)

year_cols = [str(y) for y in range(year_start, year_end + 1)]

if city_col not in df_feat.columns:

raise ValueError(f"Missing column: {city_col}")

# Temporal split definitions

split_definitions = {

'train': list(range(2013, 2020)), # Target years for training

'val': list(range(2020, 2023)), # Target years for validation

'test': list(range(2023, 2025)) # Target years for testing

}

def build_samples_for_target_year(df, target_year, window_size=3):

"""

Build samples for a single target year.

Input years: [target_year - window_size, ..., target_year - 1]

Returns: list of sample dicts and the list of feature column names used (X_cols).

"""

input_years = [target_year - i for i in range(window_size, 0, -1)]

target_year_str = str(target_year)

# base year columns (raw UER)

base_cols = [str(y) for y in input_years]

# temporal prefixes to search in df columns

temp_prefixes = ['Lag_1_', 'Lag_2_', 'Lag_3_', 'Rollmean_', 'Rollstd_', 'Diff_1_', 'Diff_2_', 'Diffmean_']

# gather candidate feature columns for the input years (only those present in df)

X_cols_set = set(base_cols)

for y in input_years:

ys = str(y)

X_cols_set.update({f"{p}{ys}" for p in temp_prefixes if f"{p}{ys}" in df.columns})

X_cols = sorted([c for c in X_cols_set if c in df.columns])

y_col = target_year_str

# Prepare arrays for all rows

X_arr = df[X_cols].values if X_cols else np.empty((len(df), 0))

y_arr = df[y_col].values

cities = df[city_col].values

# Build sample dicts (keep NaN-handling for later filtering)

samples = [

{"city": cities[i], "target_year": target_year, "X": X_arr[i].astype(float), "y": float('nan') if pd.isna(y_arr[i]) else float(y_arr[i])}

for i in range(len(df))

]

return samples, X_cols

# Build samples across target years

all_samples = []

feature_columns = None

for target_yr in range(year_start + window_size, year_end + 1):

samples_this_year, feat_cols = build_samples_for_target_year(df_feat, target_yr, window_size)

all_samples.extend(samples_this_year)

if feature_columns is None:

feature_columns = feat_cols

# Filter valid (no-NaN) samples and collect arrays

X_list, y_list, city_list, target_year_list = [], [], [], []

for s in all_samples:

if np.isnan(s["y"]) or np.any(np.isnan(s["X"])):

continue

X_list.append(s["X"])

y_list.append(s["y"])

city_list.append(s["city"])

target_year_list.append(s["target_year"])

X = np.array(X_list, dtype=float)

y = np.array(y_list, dtype=float)

# Split by target_year according to definitions

X_train, y_train, cities_train = [], [], []

X_val, y_val, cities_val = [], [], []

X_test, y_test, cities_test = [], [], []

for i, t_yr in enumerate(target_year_list):

if t_yr in split_definitions['train']:

X_train.append(X[i]); y_train.append(y[i]); cities_train.append(city_list[i])

elif t_yr in split_definitions['val']:

X_val.append(X[i]); y_val.append(y[i]); cities_val.append(city_list[i])

elif t_yr in split_definitions['test']:

X_test.append(X[i]); y_test.append(y[i]); cities_test.append(city_list[i])

# Convert to numpy arrays

X_train, y_train = np.array(X_train), np.array(y_train)

X_val, y_val = np.array(X_val), np.array(y_val)

X_test, y_test = np.array(X_test), np.array(y_test)

print("Temporal split completed:")

print(f"Training set: {len(X_train)} samples (Target years: {split_definitions['train']})")

print(f"Validation set: {len(X_val)} samples (Target years: {split_definitions['val']})")

print(f"Test set: {len(X_test)} samples (Target years: {split_definitions['test']})")

print(f"Feature dimension: {X_train.shape[1] if len(X_train) > 0 else 0}")

# Expose outputs for Orange3 Script Widget

export = {

"X_train": X_train, "y_train": y_train, "cities_train": np.array(cities_train),

"X_val": X_val, "y_val": y_val, "cities_val": np.array(cities_val),

"X_test": X_test, "y_test": y_test, "cities_test": np.array(cities_test),

"feature_names": np.array(feature_columns)

}

for key, val in export.items():

globals()[key] = val

print("Windowed samples prepared with CORRECT temporal splits. Ready for Orange3 XGBoost module.")

**Supplementary Text 3** Critical codes for generate samples (as .csv) for further temporal features construction (refer to Supplementary Text 2) to evaluate the uncertainty (95% confidential intervals) of model prediction (Method section, Figure 2a/b)

Legend: This text provides critical Python code snippets for generating perturbed samples of historical UER data, which are used to evaluate prediction uncertainty via Monte Carlo simulation. The code calculates the historical standard deviation (sigma1) of UER for each cluster (2010-2024). It then defines a function that generates multiple perturbed versions of the recent UER data (2022-2024) by adding random noise drawn from a normal distribution with mean zero and standard deviation sigma1. Each perturbed sample is clipped to a plausible range (0 to 1) and saved as a separate CSV file. These perturbed samples are used to further construction of temporal features and as input to the pre-trained XGBoost model to obtain a distribution of predictions, from which the 95% prediction intervals are derived.

import pandas as pd

import numpy as np

np.random.seed(42)

sigma1 = table1.std(axis=0)

def perturb_table2(table2, sigma1, n_samples=100):

perturbed_samples = []

aligned_sigma1 = sigma1[table2.columns]

for sample_idx in range(n_samples):

perturbations = np.random.normal(0, aligned_sigma1.values, size=table2.shape)

perturbed_values = table2.values + perturbations

perturbed_values_clipped = np.clip(perturbed_values, 0, 1)

perturbed_table = pd.DataFrame(

perturbed_values_clipped,

index=table2.index,

columns=table2.columns

)

perturbed_table.attrs['sample_id'] = sample_idx + 1

perturbed_samples.append(perturbed_table)

return perturbed_samples

n_samples = 100

perturbed_samples_list = perturb_table2(table2, sigma1, n_samples)

def save_samples_to_csv(samples, base_filename="perturbed_sample"):

for i, sample in enumerate(samples):

filename = f"{base_filename}_{i+1:03d}.csv"

sample.to_csv(filename)
